# Personal growth through navigating the world as an artist: a qualitative study of the impact of creativity camp on adolescents with depression

**DOI:** 10.1186/s13034-025-00893-6

**Published:** 2025-04-02

**Authors:** Yuko Taniguchi, Olivia Costa, Athen Ortega, Shanze Hayee, Josie Friedman, Michaelle E. DiMaggio-Potter, Jered Bright, Peng Wu, Angie P. Mejia, Gail A. Bernstein, Bryon A. Mueller, Bonnie Klimes-Dougan, Wilma Koutstaal, Kathryn R. Cullen

**Affiliations:** 1 Center for Learning Innovation, University of Minnesota Rochester, 300 University Square, 111 South Broadway, Suite 300, Rochester, MN 55904 USA; 2 Department of Psychology, College of Liberal Arts, UMN, N218 Elliott Hall, 75 East River Parkway, Minneapolis, MN 55455 USA; 3 Department of Social Work, The University of South Dakota, 4801 N Career Avenue, Sioux Falls, SD 57107 USA; 4 Masonic Institute for the Developing Brain, UMN, 2025 East River Parkway, Minneapolis, MN 55414 USA; 5 Department of Psychiatry and Behavioral Sciences, Medical School, University of Minnesota (UMN), 2312 S 6th St, Floor 2, Suite F-275, Minneapolis, MN 55454 USA; 6 School of Medicine, University of Washington, 1959 NE Pacific St, Seattle, WA 98195 USA; 7 Department of Psychology, University of Michigan, 1004 East Hall, 530 Church Street, Ann Arbor, MI 48109–1043 USA; 8 Center for Art and Dance, St. Olaf College, 1525 Campus Dr, Northfield, MN 55057 USA; 9 Research in Action, 1222 Washburn Ave N, Minneapolis, MN 55411 USA

## Abstract

**Background:**

A growing body of literature suggests that creative arts interventions can effectively support mental health and well-being in young people. We recently reported that after participating in “Creativity Camp”– a 2-week creative arts group intervention– 69 adolescents with depression showed significantly reduced depression symptoms and improved ratings of well-being. To understand the key processes impacting adolescents during and after this intervention, this study applies a multi-informant qualitative data approach.

**Method:**

Qualitative data collection methods included participatory observation notes taken during the Creativity Camp sessions and interviewing the adolescents and their parents or guardians at the end of the intervention and six months later. We analyzed data using Constructivist Grounded Theory and triangulated the findings from both sets of data to gain comprehensive and reliable interpretation.

**Results:**

We found several key processes in the adolescents’ experiences during and after camp: internal negotiation between novelty and discomfort, exploring playfulness and responsibility, discovering the uniqueness of self and others, flexible approach toward life, and an expanded view of creativity. From parent interviews, we found that their children expanded personal boundaries and enthusiasm through deep engagement, empowered perspective, and sustained enthusiasm. Triangulating the data from both sources, we constructed a theory that explains the benefits of Creativity Camp on adolescent well-being: “Personal growth by navigating the world as an artist.”

**Discussion:**

The qualitative analysis identified key processes from the Creativity Camp intervention, along with changes and long-term impacts that may have fostered personal growth. The framework of navigating the world through an artist’s lens as a pathway to personal growth presents a novel contribution to existing knowledge and practice in art-based interventions for adolescents with depression. This insight can help shape the design of future arts-in-health approaches for supporting adolescent mental health.

**Supplementary Information:**

The online version contains supplementary material available at 10.1186/s13034-025-00893-6.

## Introduction

Adolescent depression has been on the rise, with rates surging even before the COVID-19 pandemic and escalating further during and after this global crisis [1]. Since adolescence is a pivotal formative phase of development, providing appropriate and timely support is crucial to preventing long-term challenges in adulthood [2]. Standard treatments for depression, including antidepressant medications and psychotherapy, are known to be effective in some but not all [3, 4]. Furthermore, many youths with depression never receive appropriate care due to shortages of mental health professionals and other barriers, such as mental health stigma [5]. In this context, new effective and accessible strategies are urgently needed to address the current youth mental health crisis.

Emerging data have indicated that creative arts-based therapies can be effective in addressing the mental health needs of young people, both in reducing mental health symptoms and in building resilience and connection. Research on various arts-based interventions has pointed to specific health benefits, including the reduction of stress and anxiety [6–8] and improved emotion regulation [9], healthy behavior [10], and social resilience [11]. Focusing on adolescents, two rapid reviews examining the impacts of arts participation outside of school settings highlighted that creative activities can be empowering, provide opportunities to re-engage marginalized youth, and foster the development of self-esteem, resilience, and confidence [12, 13]. Considering the shortage of mental health resources and the stigma surrounding mental health challenges, arts-based interventions led by arts-in-health practitioners have been recognized as a feasible and promising option to address the gaps in the current healthcare system [14–16].

We recently developed and tested a novel arts-based intervention, *Creativity Camp*, that was designed and facilitated by artists to improve mental health and well-being in adolescents with depression [17]. The two-week curriculum was offered across eight weekdays, 4 h per day, and incorporated multiple art modalities and facilitated self-inquiry across the different art projects. Both quantitative and qualitative methods were used in the study to assess mental health, wellbeing, and participant experiences. We previously reported the results from quantitative assessments: among 69 adolescents who completed the program, depression symptoms significantly decreased, and scores on measures of well-being significantly increased after the camp, and some of these effects were sustained at six months [17]. To gain a deeper explanation of *how* the intervention led to these observed benefits, the current report focuses on the results of the qualitative methods from our study. Qualitative methods are designed to study the nature of phenomena and to help answer *why* something is observed, and thus are particularly useful in the evaluation and improvement of new interventions (especially those that are complex and multi-component) [18]. Drawing on data from in-depth interviews and participatory observations, qualitative methods provide a person-centered approach for capturing phenomena that have not previously been described, appreciated or explained, with a richness and depth that is not possible with quantitative methods [19, 20].

The qualitative data from the study included interviews with adolescents and their parents or guardians (hereafter referred to as “parents”) that were conducted at the end of the intervention and six months later, and participant-observation field notes that were made of adolescents and their interactions during the intervention sessions. Through analysis of this extensive multi-method data, we sought to identify key processes (e.g., social, emotional, cognitive, behavioral) that emerged in the adolescents’ experiences during and after Creativity Camp that appeared to be particularly impactful for their mental health and wellbeing. Through the integration of these insights, we aimed to construct an overarching theory to understand how the Creativity Camp intervention benefitted the adolescents in this study. Specifically, our interviews– which were characterized as artist interviews– focused on gaining an understanding of how the participants’ artistic experiences (e.g., adopting an artist’s perspective during the camp, and developing as an artist) may have influenced their lives.

## Methods

*Participants.* The study design and methods are detailed in a separate publication [17]. Briefly, the University of Minnesota Institutional Review Board (IRB) approved the study (IRB Identification: STUDY00014280). Adolescents ages 12–17 with depression symptoms were recruited through clinical referrals, social media and other community postings, and community engagement events. Parents completed the Child Depression Rating Scale - Revised questionnaire; adolescents with scores ≥ 12 were invited to move forward in the study. Adolescents underwent a clinical evaluation, which included a diagnostic interview and completing a series of questionnaires (see Table S1 of the Supplementary Materials for a summary of the sample’s clinical diagnoses that resulted from the interview). Before any study procedures, a parent or legal guardian provided written informed consent, and the adolescent completed an assent form.

*Setting and Structure.* The study was conducted in university spaces with spacious areas suitable for supporting day camp activities. Three cohorts were enrolled in the Summer of 2022 and three in the Summer of 2023. Each camp day involved an introduction to the project, open studio time, lunch break, and snack break (see Tables S3 and S4 of the Supplementary Materials). *Research Team.* The Creativity Camp study was carried out by an interdisciplinary team including psychiatrists, psychologists, a biostatistician, an MRI physicist, professional artists, undergraduate and graduate students, post-baccalaureate scholars, research staff, and art museum staff. The professional artists were from the local community, and were selected based both on how their artistic approach and modality would fit with the curriculum, and their experience with, and passion for, working as a teaching artist with adolescents.

*Curriculum.* The Creativity Camp intervention combines a summer day camp’s vibrant energy with an artist retreat’s immersive focus. The intervention integrates three elements that comprise high-quality summer camp experiences: supportive relationships, stimulating challenges, and opportunities to take initiative [21–23]. Simultaneously, it reflects the essence of an artist retreat by offering participants a dedicated, distraction-free environment to explore creativity, refine their skills, and connect with others in a supportive artistic community [24]. The curriculum emphasizes “thinking like an artist” through self-inquiry, art-based projects, open studio sessions, and collaborative support from professional artists. Instead of providing direct instruction, lead artists work alongside participants, encouraging mutual learning and treating adolescents as artists to foster authentic creative mindsets. This approach invites participants to experiment and reimagine their experiences, promoting novelty and self-exploration. The program encourages participants to step beyond familiar boundaries, experimenting with new art mediums and perspectives under the guidance of professional artists. These experiences nurture creativity, innovation, and personal discovery. Notably, while most camp sessions during the first three cohorts conducted in the summer of 2022 were facilitated by a primary lead artist, in the summer of 2023, guest teaching artists from the community were invited to co-facilitate several sessions. Further curriculum details are provided in Table S2 of the Supplementary Materials. In May 2023, adolescents from the first summer were invited to showcase their artwork in a public art exhibition at the Weisman Art Museum on the University of Minnesota campus.

### Qualitative data collection

As suggested by the Consolidated Criteria for Reporting Qualitative Research [25], we first report the researchers involved in the data collection: (1) Participatory observation note takers (also referred to as near-peers), who were undergraduate students and post-baccalaureate scholars observing adolescent participants while themselves actively engaging in all camp activities, and (2) interviewers, which included participating researchers, staff, and co-investigators (see S10 for more information about the research team).

*Participatory observation notes.* During and after each session, participatory observation note takers recorded field notes to document their observations of the adolescents’ behaviors and interactions. A detailed protocol for the participant observation procedure is provided in S10 of the Supplementary Materials. In brief, participatory observation note takers jotted brief notes throughout each session, consulting with one another at the end of each day to compile detailed descriptions of their observations. The semi-structured field notes focused on key aspects such as engagement levels, participation, emotions, attitudes, and social interactions.

*Adolescent and parent interviews.* On the final day of the intervention, adolescents were individually interviewed about their artwork and overall experience. Framed as an artist interview, the purpose was to gain insight into adolescents’ experiences of creating artwork and the meaning behind their creations. Since the artwork participants had developed throughout the camp focused on self-inquiry and reflection, the interviews remained open-ended, following the principles of grounded theory, which emphasizes an open, data-driven approach. We did not explicitly ask about depression or mood for this interview since these clinical domains had been assessed using quantitative assessments [17]. Rather, the interviews were designed to explore how participants’ past experiences (including those related to depression) may have shaped their artistic expression and how they felt during the creative process about the artworks they had created. In contrast, we interviewed parents immediately after the camp to gather their insights and observations on the intervention’s impact on their children. In these interviews, we explicitly asked about any changes in their children’s initiative to be more social or physically active, as these factors are closely related to mood shifts and depression. Both adolescents and parents were again interviewed six months later to assess the long-term impacts. Interviews were conducted by research team members including near-peer mentors (OC, SH, JF, MD), investigators (YT, PW, AM, GB, BK, WK, and KC) and staff. The training process (led by AM) for conducting the interviews included both video instruction and in-person practice interviews. Since all interviews happened simultaneously, a large team of interviewers was needed. Interviewers and interviewees were paired based on participants’ comfort levels observed during the camp. For instance, shy participants were matched with near-peer interviewers they already knew to create a more comfortable environment, while adolescents deemed to be more confident or relatively more at ease were paired with staff or co-investigators whom they had not previously met. All interviews were recorded and transcribed for qualitative analysis, and analyzed qualitatively. While interviews in the summer of 2022 were conducted in person, interviews in the summer of 2023 were conducted over video conference. Interview questions are shown in S6-S9 of the Supplementary Materials.

### Qualitative data analysis

*Methodology.* We employed a Constructivist Grounded Theory (CGT) methodology [26], which recognizes that data and analysis are co-constructed through interactions between researchers and participants [27]. CGT is particularly valuable for identifying and describing phenomena not well-understood or not yet fully articulated in existing literature. Given that Creativity Camp is a novel intervention, CGT’s inductive methodology was especially well-suited to our research goals. This approach enabled us to generate a theory grounded in adolescents’ experiences while cautiously applying theoretical interpretations. While the adolescents’ perspectives were considered the primary data source, we integrated insights from parents’ views and perspectives based on their observations of their children outside of the camp context. Our data analysis followed the iterative and systematic CGT coding process, comprising four stages: initial coding, focused coding, axial coding, and theoretical coding (see Table 1).

**Table 1:** CGT analytical process

Initial coding	Conduct segment-by-segment coding to separate the data into smaller units and capture a wide range of actions, meanings, and expressions while remaining closely tied to adolescents’ experiences. Aim to retain all possible interpretations without imposing preconceived categories.	Throughout the process: Engage in memo writing to document analytic decisions and the procedure (or process) of constant comparison [28], iteratively compare codes, categories, and data to refine our understanding and ensure that the analysis remains grounded in data.
Focused coding	Identify and group the most significant initial codes to begin forming broader conceptual categories. Refine codes by eliminating redundancy, merging conceptually similar ideas, and identifying gaps that need further exploration.	
Axial coding	Explore relationships between categories, subcategories, and contextual factors and examine the conditions, actions, and consequences associated with the central phenomena. This allows us to deepen our understanding of how various factors within the Creativity Camp environment and beyond influenced participants’ experiences.	
Theoretical coding	Integrate core categories into a cohesive framework to construct an emerging theory and identify the central phenomena that connected and explained the data.	

**Fig. 1:**
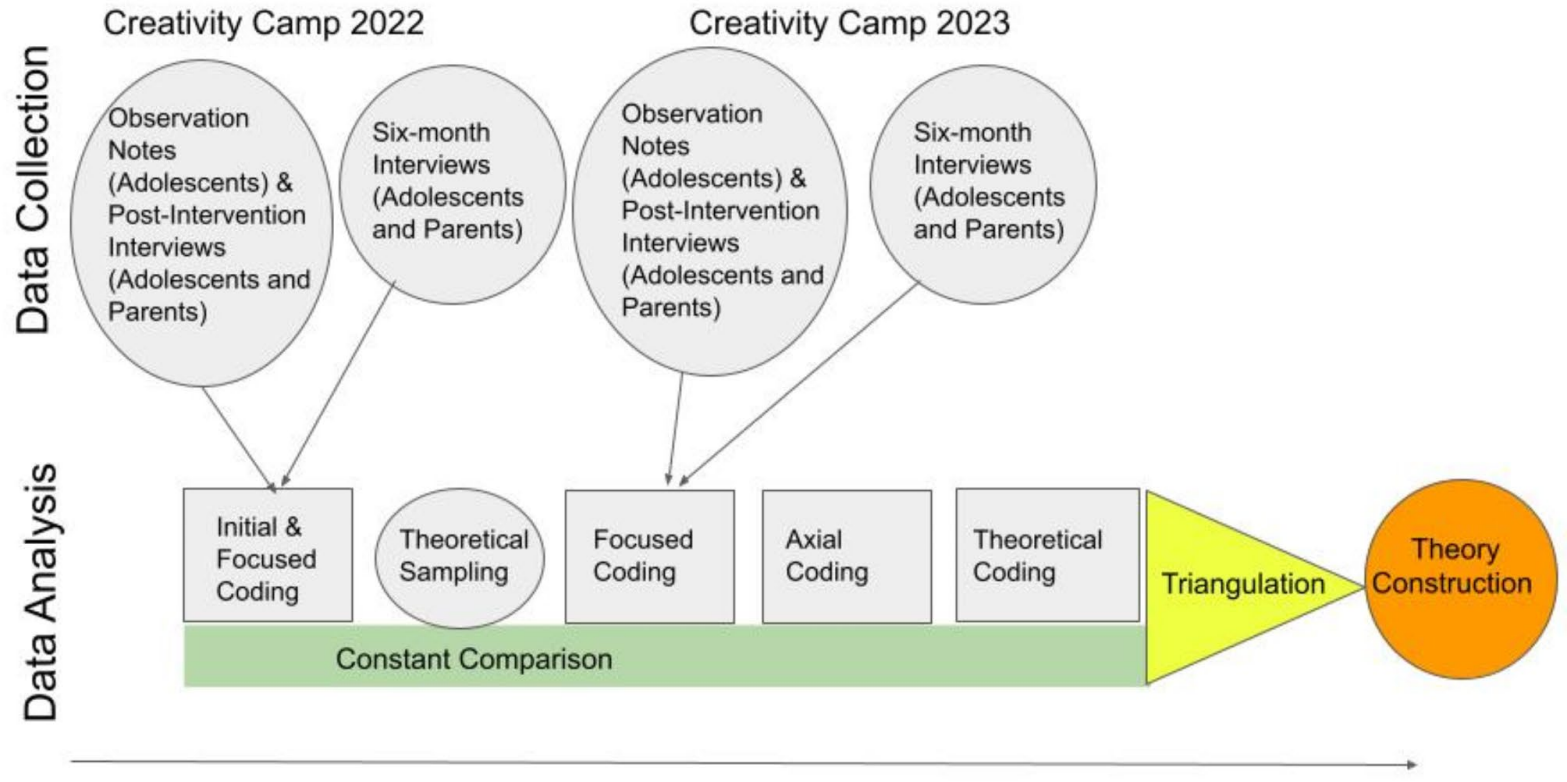
CGT data analysis overview

Figure 1 provides a graphical overview of the entire analysis process, including the data triangulation step described below.

*Data analysis team and process.* Initial coding was conducted by a team of 20 undergraduate students (who had not been a part of the Creativity Camp intervention or data collection) under YT’s supervision. The initial and focused coding was conducted primarily by these “intervention-naive raters.” Members of the research team who had participated in the camp or interviews contributed later, mainly during the axial and theoretical coding phases, when their contextual knowledge was most relevant and applicable. Data coding was performed independently and then collaboratively reviewed and discussed to achieve agreement among the researchers and enhance the findings’ credibility. Disagreement about the codes was resolved using the open discussion strategy [29]. The researchers’ background in Psychology (OC, JF, SH) and Health Sciences (AO, JB), combined with the lead researcher’s expertise in Arts in Health (YT), added an extra dimension of reflection to methodological choices and the credibility of conclusions while ensuring the findings reflected the participants’ perspectives rather than the researchers’ expectations [30]. (See S10 for additional information on the research team.)

*Adolescent-focused data (observation notes and interviews).* Using the CGT method described in Table 1, coding, constant comparison, and memo-writing began with the data from the first cohort in 2022. The coding team then applied these initial codes when analyzing the data from the new participants of the summer of 2023, integrating the new information while synthesizing and identifying patterns and relationships from the earlier coding and assessing theoretical saturation [31], following the process described in Table 1. The six-month interviews also followed the same analytic process, specifically emphasizing exploring the link between adolescents’ experiences in Creativity Camp and long-term impact.

*Parent interviews.* Coding of the interview data using the CGT method began after the completion of the 2022 Creativity Camp cohorts. Similarly to the adolescent data analysis, the focused coding schemes were adjusted when the 2023 interview data were added. The six-month interviews were added for further focus coding.

*Theoretical sampling.* Between 2022 and 2023, the interview questions were adjusted to respond to curriculum updates and informed by the team’s assessment of the theoretical saturation [31] they observed from their analyses of the data from 2022. (In a grounded theory study, theoretical saturation occurs when additional data no longer yield new insights, themes, or categories relevant to the study.)

*Triangulation.* A triangulation process [32] was employed to integrate findings from adolescent-focused data (observation notes and interviews) and parent interviews. This process aimed to deepen the understanding of adolescents’ experiences and assess how the different perspectives of these experiences were shared and interpreted across the data sources. Parents’ perspectives were particularly valuable, offering insights grounded in their knowledge of their children and the changes they observed. This approach enriched the analysis by providing a holistic view and strengthened the findings by validating them across diverse data sources, ensuring reliability and depth [33].

*Theory construction.* Using storyline strategies [34], we translated the theory into a coherent narrative. The final theory was drafted, revised, and refined through iterative team discussions, ensuring it evolved into a story of insights while remaining true to the data.

## Results

*Participants.* During the summers of 2022 and 2023, in 6 different cohorts (3 per summer), 77 adolescents were enrolled in the study and completed the baseline interview. Of these, 69 completed the Creativity Camp intervention, including the interview on the last day of the intervention, along with 69 parents who completed the post-intervention interview. A total of 65 adolescents and 65 parents completed the 6-month interview. Table S1 in the Supplementary Materials provides demographic and clinical characteristics at baseline.

### CGT results from adolescent data (participatory observation notes and interviews)

**Fig. 2:**
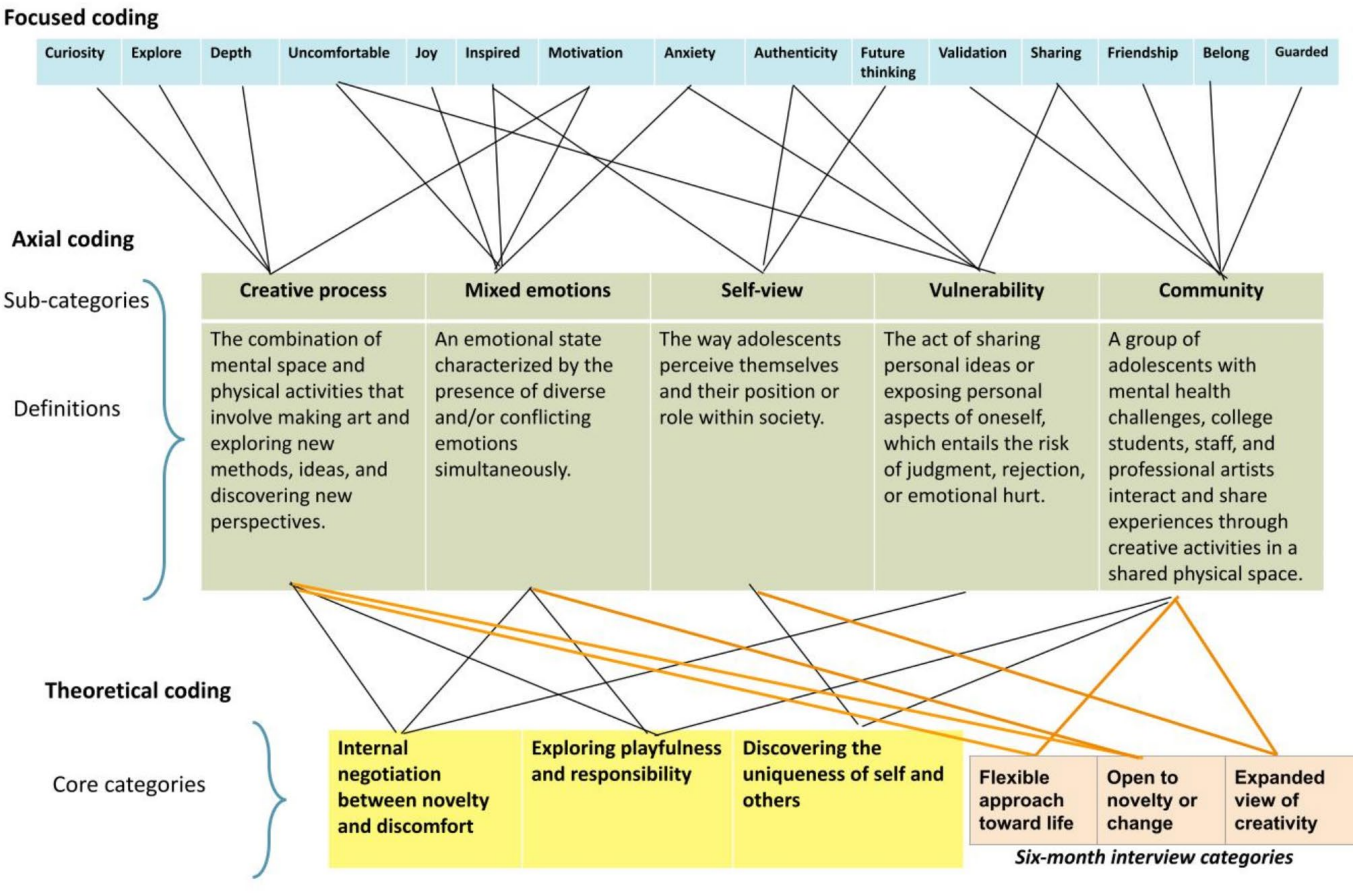
Adolescent participant data coding process overview

From the codes listed in Table S5 of the Supplementary Materials, the team constructed five subcategories, “Creative process,” “Mixed emotions,” “Self-view,” “Vulnerability,” and “Community.” The interplay and synthesis of these subcategories led to the construction of three core categories. Figure 2 demonstrates the construction process from focused coding to theoretical coding.

In the following sections, we demonstrate how each core category was constructed using representative quotations from adolescent interviews. To protect participant confidentiality, all pronouns in example quotes have been standardized to “they/them” and names are omitted.

#### Core category 1: internal negotiation between novelty and discomfort

This core category was constructed through the interplay of three subcategories. “Creative process” reflects adolescents’ engagement in art-making, where they experience both the excitement of novelty and the discomfort of ambiguity, as represented in “Mixed emotions.” Expressing new ideas involves taking risks, such as presenting potentially inadequate concepts and navigating higher personal stakes, captured in the subcategory “Vulnerability.”

Many adolescents encountered novelty during the camp, which sparked curiosity and excitement alongside discomfort and apprehension. This often led to an internal negotiation about interpreting and responding to their experiences (i.e., feeling discomfort in the face of novelty and trying to decide between approach versus avoidance). In this circumstance, adolescents commonly expressed feeling “uncomfortable,” “anxious,” and “exhausting,”; meanwhile, other kinds of sensations like “fun,” “surprising,” and “exciting” served to instill the momentum of their Creative process. Internal negotiations occurred at various points, from deciding to attend the camp each day to selecting which ideas, thoughts, and emotions to express. The outcomes of these negotiations ranged from feelings of bravery to ambivalence. Two quotations from adolescent interviews exemplify this:

“I wanted to participate in everything as best I could. Then right before [the camp], I was like, I don’t know if I want to do this. I’m glad I did cause the camp was way better than the beginning surveys. The second day of the big painting was very exciting. When I got home, I was like I wanna go back. But all other days, I was like, I don’t wanna wake up [early] cause it was hard.”

“It’s despair because of the meaning of what it [my artwork] is and the sadness around it. But I’m also proud of myself because I’m putting it [sadness] into an art form and I am working really hard on it. I guess I like doing something about the feelings that happen.”

This core category highlights adolescents’ internal processing as they grappled with and articulated their complex thoughts and emotional responses. Additional illustrations of this core category are detailed in Table S14 of the Supplementary Materials.

#### Core category 2: exploring playfulness and responsibility

This core category was constructed from the subcategories “Creative process,” “Mixed emotions,” and “Community,” capturing how adolescents adopted a playful approach to exploring and encountering new ideas while also taking responsibility for their choices and working hard to bring their unique creative vision to life.

An illustration of the process of exploring playfulness and responsibility came from an adolescent who described:

“I was making some random blue background, and I was going to [paint] some cool clouds and grass and the sky. I didn’t like that, so I started adding random colors. As I was adding random colors, it started being really pretty. Then I started adding white, and the artist dude [guest artist] suggested adding baby blue.”

This same scene, observed by a researcher, shows this participant’s playfulness and emerging responsibility as an artist. The researcher noted:

“They were really excited about the big painting, but they weren’t sure how to approach this activity. We [the research assistants] asked them what they felt passionate about. They said that they weren’t sure. Then they said that they really love their mom so we suggested they represent their mom without actually painting their mom. We asked them what their mom’s favorite color was, and they responded, ‘I hate my mom’s favorite color, and I don’t want this to be that!’ Eventually they started painting, was done really quickly, and walked around for a bit. We asked them what we should include in our painting and if they could give us inspiration. They said, ‘flowers.’ When they came back to look at the flowers, they said, “THAT IS GIVING” really excitedly. We noticed them observing us talking to the guest artist about our pieces and what else we should add or do. A few minutes later, they asked the guest artist the same thing. They started to get to work again on their piece 15–20 min before we had to wrap up.”

**Fig. 3:**
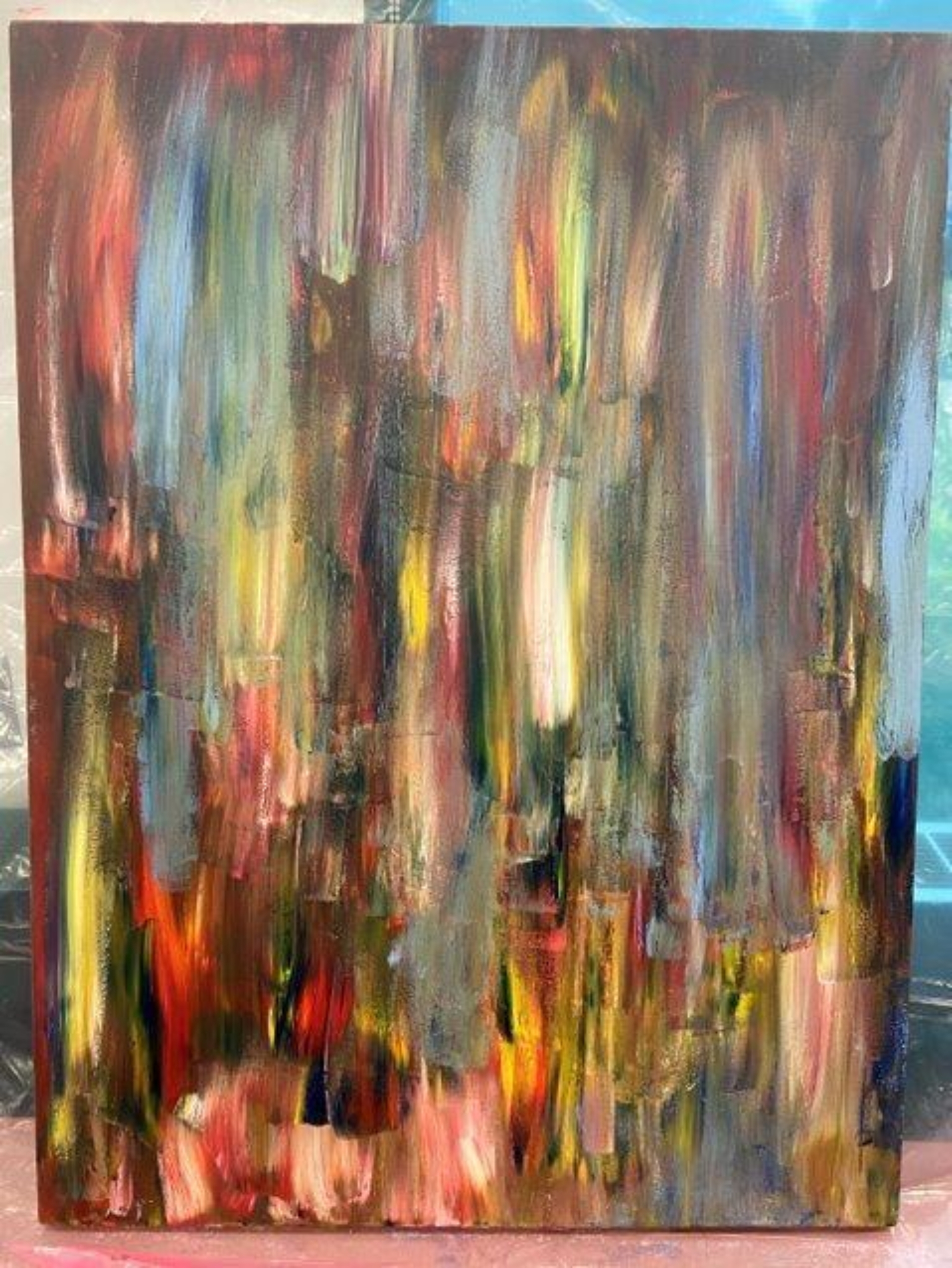
Abstract painting by a participant

This observation describes their initial exploration as “random.” Still, they worked toward realizing a vision that, through experimenting with various colors and guided by feedback, felt artistically and intuitively “right” and then worked hard to see it through (see Fig. 3 for the final product). Additional illustrations of this core category are detailed in Table S14 of the Supplementary Materials.

#### Core category 3: discovering the uniqueness of self and others

This core category was constructed from the subcategories “Self-view” and “Community,” highlighting how we develop a new awareness of ourselves and others when placed in a unique context, a community where adolescents, college students, staff, and professional artists come together and connect through creative activities in a shared physical space. Many adolescents were surprised by the unique qualities in both their work and that of their peers. They gained insight into the reasons and processes behind each individual’s creative decisions, realizing that their creations were inherently authentic and could not be replicated by others. This recognition that what we create is naturally unique was reinforced daily through witnessing the creative process in the Creativity Camp community. In an interview, one adolescent reflected:

“I’ve just noticed that I have a lot in common with people but also [I’m] extremely unique. It’s like a uniqueness I didn’t quite understand, but it’s just powerful I guess to understand that like, ‘Wow, I really am the only one of me,’ and that’s always kind of been a hard thing cause I never felt like I fit in but then I realized that I didn’t really have to [fit in]. It’s not a requirement. They [other people] are different from me but also how they can be so similar at the same time.”

Although all adolescents were given the same prompt, guidance, and materials, the creative outcomes that emerged markedly diverged, leading to the generation of uniquely individual artworks. This outcome provided tangible evidence for adolescents to recognize and appreciate how individuals can be similar and yet also distinct, shaped by their specific perspectives and life experiences. Additional illustrations of this core category are detailed in Table S14 of the Supplementary Materials.

#### Long-term impact

The six-month interviews reveal that many adolescents continued to navigate discomfort while searching for creative experiences and incorporating playful exploration into their lives. Two additional core categories emerged from these follow-up interviews: (1) flexible approach toward life, (2) open to novelty or change, and (3) an expanded view of what it means to be creative.

*Flexible approach toward life.* This category was constructed based on the subcategories “Creative process” and “Community.” Many adolescents described how the camp influenced them to think, notice, feel, and explore their lives differently; they also reported that they pursued new opportunities in school and navigated difficult situations, exemplifying a notable sense of flexibility after the camp. For instance, one adolescent auditioned for two theater roles but was not selected. Reflecting on the experience, they remarked, “But at least I auditioned,” viewing the outcome as evidence of their willingness to take on challenges rather than automatically labeling it a failure. Adolescents often reported navigating uncomfortable situations with an open and adaptable mindset, recognizing that the outcomes were yet to be determined:

“[After the camp, ] I got into art and doing it more especially since I spent two weeks working on stuff. I took a pottery class my first semester. And I kind of became friends with the guy who sat next to me just because we were kind of similar. We were both uncomfortable. I was like ‘why am I taking this class?’ And then we ended up both really liking it, and we kind of helped each other on projects.”

Instead of deciding that taking the class was a mistake, the adolescent demonstrated flexibility by remaining open to different possibilities. Together with the new friend, they “helped each other” as they negotiated between novelty and discomfort until they “ended up” encountering a positive outcome. Trying new things involves risk and discomfort. Adolescents demonstrated an ability to stay in the situation with a flexible outlook.

*Openness to novelty or change.* This category was constructed based on the subcategories “Creative process” and “Mixed emotions.” Additionally, more than half of the adolescents noted an increased willingness to try new things and felt that the camp inspired them to continue exploring creativity in their lives. Several adolescents noted how they *allowed* exploration without a clear idea of the outcome:

“Because I went to the camp, I’m more flexible. I make a lot of stuff at home, I like sharing it, and I just kind of make stuff up. Recently I was just drawing in my notebook. Then I made this whole thing that I just came up with, this whole murder mystery; each page you turn, you get new clues. That was fun.”

Table S14 of the Supplementary Materials details specific examples of flexibility and openness, demonstrated through adolescents’ willingness to fully engage with different ways of thinking and feeling and embrace both the freedom and risks posed by all creative endeavors.

*Expanded view of what it means to be creative.* This category was constructed based on the subcategories “Self-view” and “Community.” Reflecting on their Creativity Camp experience, many adolescents expressed a newfound understanding that creativity extends beyond generating novel ideas or engaging in art. It also involves noticing subtle moments and viewing ordinary concepts innovatively. With this realization, many adolescents began to see themselves as creative beings:

“[After the camp] I notice myself thinking just more about things. I wasn’t a very creative person, and I didn’t really think of things that way. [But] I did think [creatively] for most of the camp. It’s hard when you’re around people that are super into art. That was just not for me. But I do notice myself more now, like thinking about things and being more interested in art. I try not to compare myself when it comes to art because it’s entirely subjective.”

“I discovered that I have a lot of potential and a lot of creativity that I definitely didn’t know of beforehand. And definitely coming to the camp, it made me see that there’s a lot of different ways to be creative. It’s not just art. There’s like a ton of other ways. So that definitely showed me that there’s a lot more.”

Through this expanded understanding of what creativity means and who can be considered creative, some adolescents noted that they now “care more” about topics they previously found uninteresting or have come to recognize that potential materials for artistic and creative opportunities exist around them. These reflections underscore how adolescents broadened their perspective on creativity, gaining insight into what inspires their creative process, learning to appreciate subtle moments they once overlooked, and ultimately influencing how they view themselves as creative individuals. Specific examples of this category are detailed in Table S14 of the Supplementary Materials.

### Results from parent interviews

**Fig. 4:**
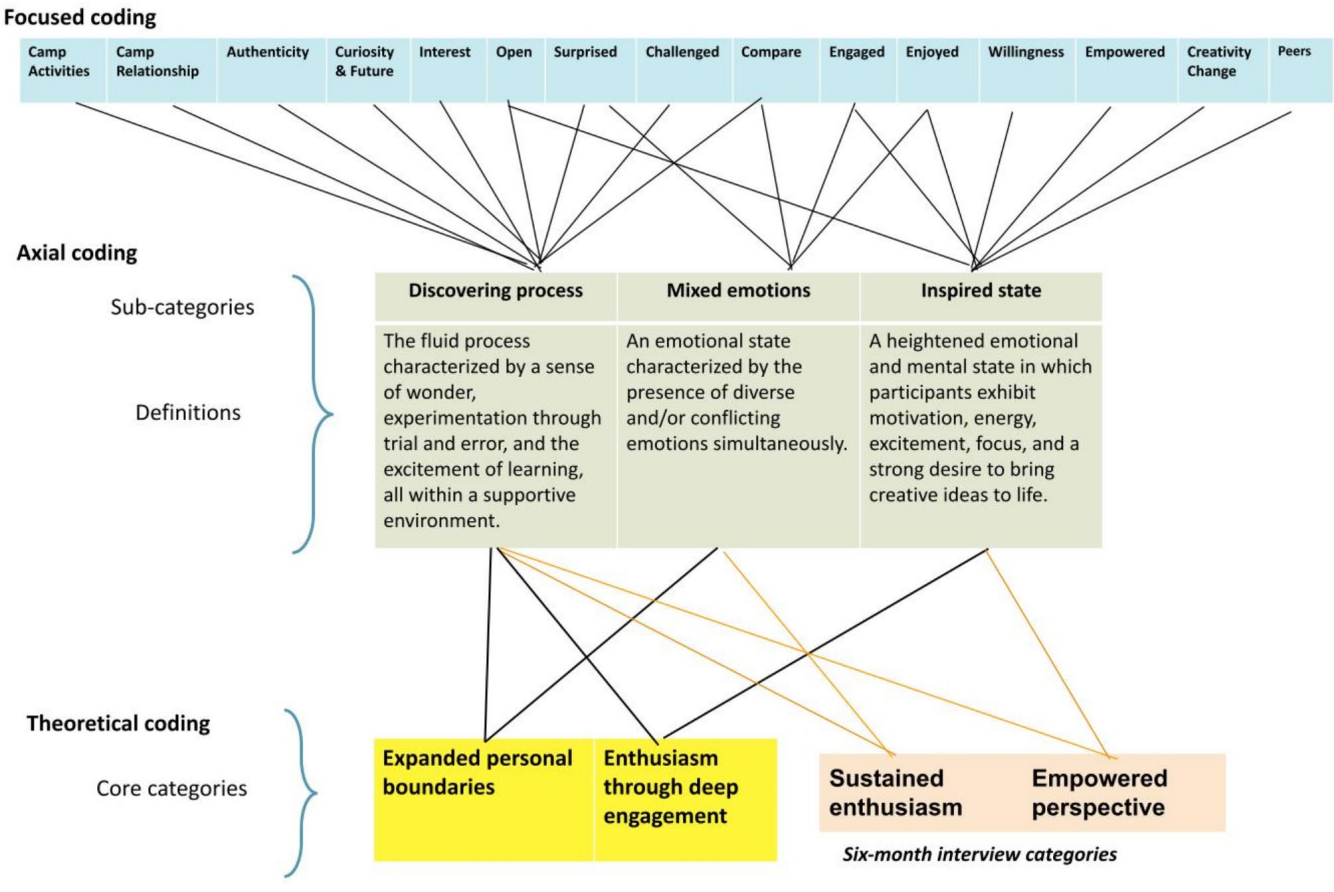
Parents/guardians interview data coding process overview

From the codes listed in Table S13 of the Supplementary Materials, the team constructed three subcategories capturing the themes from the Parent Interviews: “Discovery process,” “Mixed emotions,” and “Inspired state.” The interplay of these subcategories led to the construction of two core categories: Expanded personal boundaries and Enthusiasm through deep engagement. Figure 4 demonstrates the construction process from focused coding to theoretical coding of parents’ interviews.

#### Core category 1: expanded personal boundaries

This category was constructed from the subcategories “Discovery process” and “Mixed emotions.” Parents frequently observed how their children’s actions exceeded their normal boundaries and comfort. For instance, one of the activities, which involved dance, challenged one adolescent who, in addition to depression symptoms, also had obsessive-compulsive disorder (OCD). Their parent reported:

“One day, they said they danced and said, ‘you’re not gonna believe it. I even touched hands with a lot of people.’ They can’t really touch [others] honestly because of their OCD. They don’t accept a lot of that [physical touch.]. So we had a very specific conversation about that. And I was super proud of them for getting out of their comfort zone, doing something that they normally would never do.”

The parent indicates how their child’s action was unexpected, and points out that they were in an environment in which they navigated through discomfort beyond their customary boundaries.

Outside of the creative activities, many parents reported their children’s curiosity and excitement about the culturally diverse lunches at camp that were intentionally included to provide additional novelty and variety. Even parents of adolescents with complicated relationships with food reported that they were surprised their child not only tried but enjoyed the exploration and discovery around the lunches. For example:

“Very surprisingly, they enjoyed the variety of food. This is extremely surprising because they won’t even eat anything I make. And I’m their mother. They are so paranoid about what people are going to put in it. They can’t control how much oil is used. They can’t control the ingredients, therefore the calories, therefore anything… Their problems are eating disorder-related. It was a different ethnic food every day, but they really enjoyed that. They talked about that a lot.”

Parents also observed that such expansion of personal boundaries extended to the home:

“I noticed they’re ordering things from places they normally wouldn’t order.”

“[The adolescent] liked Thai food, Indian curry, and some Japanese food. They thought their brother or step dad would [also] like what they liked. It did spark some conversation.”

Initiating such discussions and offering suggestions about family members’ preferences were beyond their usual behavior from the parents’ perspectives. Specific examples of this category are detailed in Table S16 of the Supplementary Materials.

#### Core category 2: enthusiasm through deep engagement

This core category was shaped by parents’ perspectives, captured in the subcategories “Discovery process” and “Inspired state.” Many parents described their children as engaged, excited, and energized during the weeks of the camp, even at home:

“They’re just excited overall. Even going to therapy [they were] very animated and excited. They had a lot of positive things to talk about and focus on. They’re more positive in our communication with family.”

**Fig. 5:**
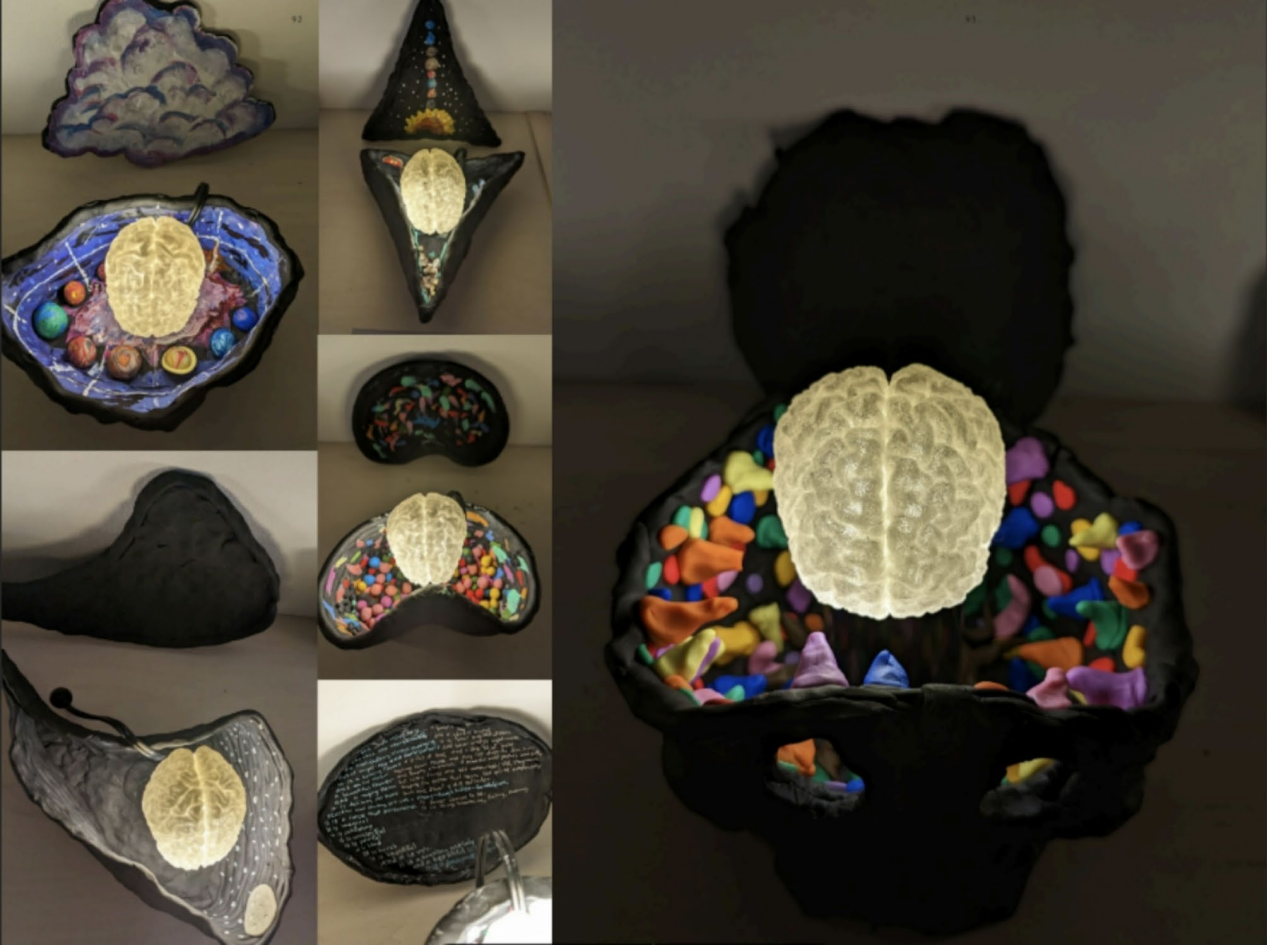
Clay boxes participants made for their 3D brain models

Parents also noted shifts in their children’s interests. For example, in one of the camp activities of 2022, adolescents received a 3D model of their own brain (generated from an MRI assessment of the study) and then created a clay box for the model (see Fig. 5). This ignited curiosity and interest in science:

“They have always been a little interested in science but [the 3D brain model] grabbed them enough to want to do something about it. They brought it home and showed us all. They were just delighted and said ‘this is what is in my head, making things happen, and it’s not like anyone else.”'

Many parents noted that their children’s positive mood and overall enthusiasm were unexpected; instead, they expected their children to resist going or lose interest in attending the camp based on their children’s previous tendencies. Considering that the camp was eight days, parents were surprised that their children did not discontinue the program:

“They certainly seemed very engaged. They got up on time for every session, and that is extremely unusual.”

“It just really struck me from the first day and every subsequent day that they would just come home looking and feeling or just the way they held themselves unburdened or light in a way that, you know, certainly doesn’t happen after a day at school. So and certainly was different from when they left to go to the session. So that was just great to see.”

The parents’ surprise suggests that, to some extent, they had anticipated their children might lose interest or motivation over time. However, they interpreted even subtle actions, such as waking up on time or discussing camp experiences, as clear indicators of their children’s enthusiasm for Creativity Camp. Specific examples of the enthusiasm parents identified as an unusual trait in their children are detailed in Table S16 of the Supplementary Materials.

#### The long-term impact of the camp based on parents’ observations

Based on the six-month parent interviews, two categories were identified: (1) Sustained enthusiasm and (2) Empowered perspective.

*Sustained enthusiasm.* This category, informed by the “Discovery process” and “Mixed emotion” subcategories, demonstrated that their children remained enthusiastic and energetic, primarily through engaging in creative activities more at home and in school. This change also included new activities, as parents described that their children “picked up a drum” and “were inspired to do a bunch of spray painting artwork and made some stuff for the house.” Examples of the enthusiasm parents identified as an unusual trait in their children are detailed in Table S17 of the Supplementary Materials.

*Empowered perspective.* This category, informed by the subcategories “Discovery process” and “Inspired state,” characterized a mindset in which individuals felt open and motivated to take action with an awareness of their power and abilities. Many parents described that their children demonstrated open-mindedness, with less polarized views, and perhaps a more trusting approach to the world, leading to exploring new experiences. For example:

“It’s been really nice to see them more open to new experiences and going places that they wouldn’t have gone before. It’s not even just with artistic stuff. It’s anything that they haven’t done or people they haven’t talked to. It’s like they can now see that it could end up being a good thing. Versus before I think everything was just really negative, like they couldn’t see that side.”

In addition, many parents observed that their children actively created opportunities to lead, initiate, and develop experiences for themselves or others despite ongoing challenges with depression. In the context of these more empowered perspectives, parents reported their children were more willing to explore new therapies or medications. In addition, parents noted their child’s deep engagement in Creativity Camp might have offered a valuable example of community and structure that their children appeared to replicate in their own way. For example:

“A couple of the kids they did Creativity Camp with, they kept as friends which is great. They still keep up with them, and the nice thing is that they normalized the understanding of having challenges and dealing with depression. I think that’s been a huge factor because there is a difficulty when you are the one struggling with mental illness and you perhaps have an existing cohort who cannot empathically understand what that entails […] now, they [my child] has become a mentor for a lot of the other kids at their school. The school has commented that they have their own cohort. It’s all younger kids who are having trouble fitting in usually through mental illness or family situations that are beyond their control. When at school, they are really able to work with these kids a little bit or at least provide them with some emotional support, which has been fantastic.”

In addition to the empowered compassion for other peers described above, parents observed their children communicating more compassionately with their siblings at home.

### Triangulation

Through the triangulation process, the integration of adolescent and parent data findings revealed a strong alignment between the core categories and long-term impact from both datasets. Analysis of parent interviews confirmed and extended the core categories identified from adolescent data. At six months, the alignment between the themes that emerged from the adolescents’ data (flexible approach, expanded view of creativity) and those that emerged from the parents’ interview data (sustained enthusiasm and empowered perspective) supports a cohesive narrative of the adolescents’ personal growth. Adolescents demonstrated courage and a flexible approach while reflecting on their willingness to stay in discomfort and engage in unfamiliar activities. Similarly, parents heard about their children’s experiences and observed this openness and their children’s eagerness to try new things at home, even in the months following the camp. Sustained enthusiasm observed by parents further connects the two datasets. Adolescents reported continued exploration of creative endeavors six months after the intervention, demonstrating a persistent curiosity and engagement with their artistic and personal growth. This report aligns with parents’ observations of their children maintaining sustained enthusiasm and interest, as evidenced by their initiative to try new activities.

### Findings from the triangulation process

**Fig. 6:**
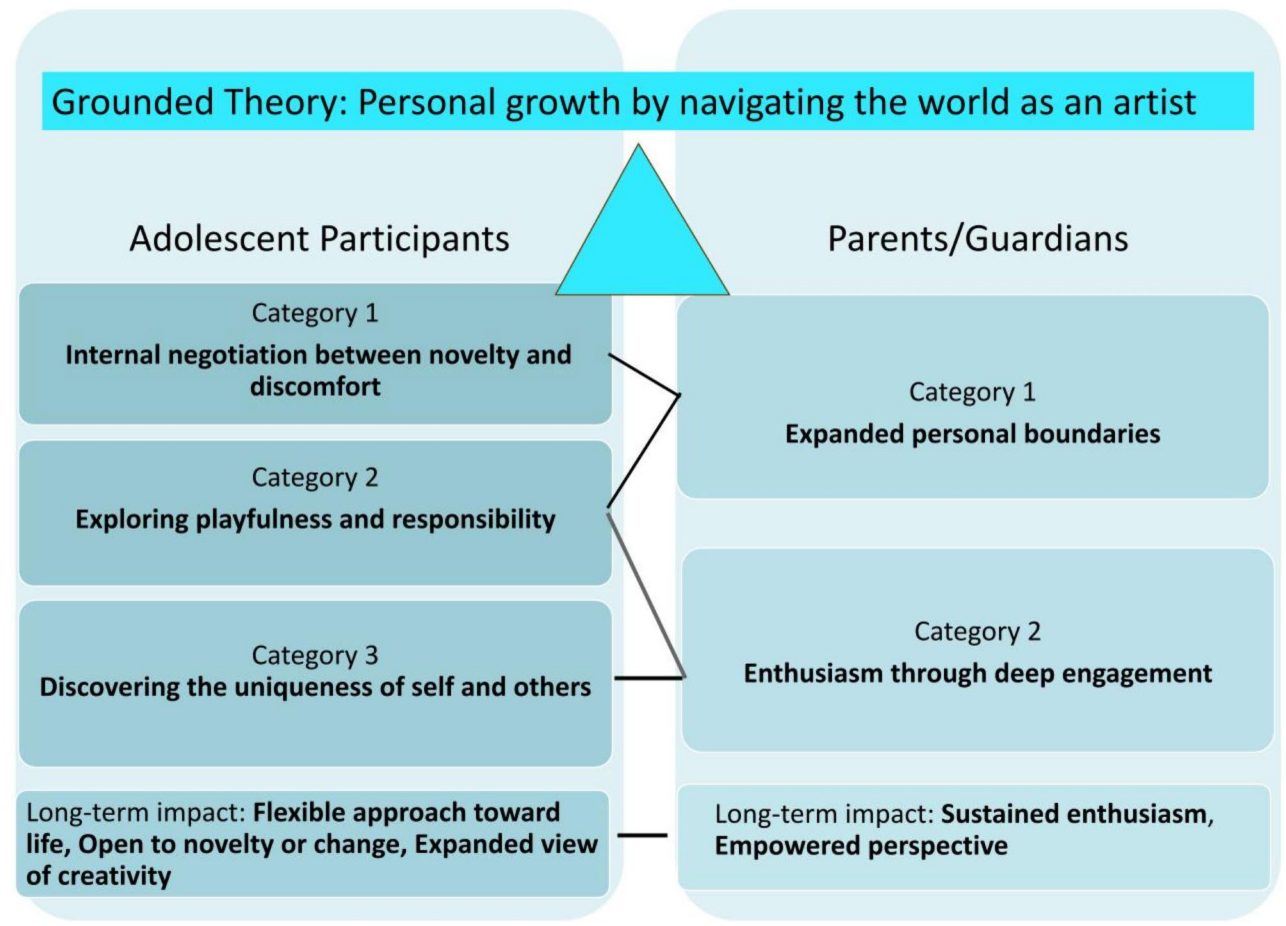
Connecting core categories to grounded theory

Following the triangulation process, and based on the convergence of findings, we constructed the theory, “Personal growth by navigating the world as an artist,” to understand the impact of Creativity Camp on the adolescents in the study. Figure 6 graphically summarizes our theory, incorporating the many varied but convergent findings from the adolescents and parents both during and soon after the Creativity Camp and at the six-month follow-up.

The concept of “navigating the world as an artist” reflects the expanded perspectives through which adolescents had begun to view their own world based on their insightful experiences. The results suggest that negotiating between novelty and discomfort, embracing playfulness and responsibility as artists, and recognizing the uniqueness of themselves and others in the Creativity Camp environment through diverse learning opportunities contributed to personal growth. This growth is evident in the six-month interviews, where adolescents describe new ways of connecting with their life activities and environment compared to their experiences before the camp. While the activities and environment remained unchanged, adolescents reported a shift in their perception and interpretation of their lives. This signifies an emerging understanding that using their creativity involves exploring multiple possibilities rather than adhering to rigid pathways, highlighting their personal growth and a transformative change in mindset. Many adolescents said they realized there are “no right and wrongs in creativity.” This openness, which is required of an artist, influenced how they approached their lives after the camp.

Notably, similar signs of personal growth emerged from both the adolescent and parental data sources. We did not find significant discrepancies between the comments and perspectives of participants and their parents. Most parents were well aware of their children’s experiences and acknowledged them in similar ways. However, while there were no major discrepancies, we did find some important complementary insights in a few cases where parents were able to elaborate on the reasons behind their children’s perspectives. For example, in one specific case, a child reported not having a great time at camp, feeling disconnected from peers, and unengaged in creative activities. The parent, however, explained that the child’s depression often makes it difficult for them to enjoy experiences. Despite this, both the child and parent chose to continue with the camp. By the end, the child still described the camp as a challenging experience, and the parent acknowledged this but maintained that the experience was ultimately beneficial. As this example illustrates, while adolescents share their firsthand experiences, parents often provide an analytical perspective that adds context. These differences are not discrepancies but rather complementary insights that deepen our understanding of the findings.

## Discussion

To provide a more in-depth understanding of adolescents’ experiences during and after the Creativity Camp intervention, this study used qualitative data from multiple sources (adolescents, parents, participatory observation note takers) to identify the key processes, changes, and long-term impacts that may have contributed to the significant clinical improvements in mental health and well-being that we previously reported [17]. The triangulation process to integrate the themes from the various sources led to a unifying theory, positing that Creativity Camp leads to “Personal growth by navigating the world as an artist.” Building on emerging theories and interventions that have begun to focus on personal growth as an approach to helping patients navigate depression [35–38], the concept of navigating the world as an artist to achieve personal growth for adolescents with depression represents a novel contribution to the field. In the following paragraphs, we discuss the main concepts that generated this theory, reflecting on their relevance to depression and developmental processes in adolescents.

*Internal negotiation between novelty and discomfort.* Adolescents must respond to competing drives as they move through the many challenges they face. Pursuing something novel that is exciting but also scary holds both risk and potential reward. Mental health disorders such as depression and anxiety impact the motivational drives of approach and avoidance [39], including in children with depression [40]. Adolescents with depression tend to avoid novelty and discomfort, many times dropping out of activities, staying at home, and experiencing an increasingly narrower version of their lives. Accordingly, parents noted surprise at their children’s consistent program attendance, which signaled an increase in their motivated behavior. “Thinking like an artist” requires testing out novel experiences even when they are uncomfortable. In this way, wearing the “artist hat,” adolescents practiced pursuing novelty despite the discomfort. Our findings add to prior qualitative research studies that have also identified elements of discomfort and boundary exploration in creative arts interventions for adolescents with mental health disorders. For example, a review of three quantitative and ten qualitative studies on creative art therapies for adolescents with eating disorders [41] found that, across studies, participants experienced conflicting emotions such as anxiety and fear; a heightened awareness of their boundaries; and empowerment in navigating these emotions and boundaries by using available resources and making decisions for themselves.

Follow-up data from adolescents and parents showed how adolescents translated this practice to other aspects of their lives, evidencing sustained personal growth. The *Expanded personal boundaries* theme provides additional evidence of the adolescents’ abilities to push against the tendency to avoid situations that typically cause discomfort, to pursue what’s important to them. In their six-month interviews, adolescents shared a pattern of incorporating flexibility in approaching challenges. In depression, patients can fall into persistent, “stuck” thought patterns that are habitually negative and repetitive [42]. Such habitual thinking patterns can predict relapse after recovery [43]. Thus, the practice learned in Creativity Camp may have contributed to sustained well-being.

*Exploring playfulness and responsibility.* This artistic development process addresses two key areas of depression symptoms: playfulness introduces fun/enjoyable experiences in the face of low enjoyment (anhedonia), and responsibility fosters a sense of mission and purpose in the face of hopelessness. Play is a critical aspect of childhood [44, 45], but many adolescents have few play opportunities by adolescence. Researchers have previously connected the “decline of play” in North America in recent decades to an increase in child and adolescent psychopathology [46]. Creativity Camp’s open studio structure allowed adolescents to explore and play with new materials and ideas. They received the freedom to play, to experiment, and to explore, allowing them to create their artistic visions. Many adolescents described how this differed from other, stricter settings in their experiences. They decided whether/how much to engage in activities and when to take breaks. They chose how to begin, proceed, and complete their artwork. As one adolescent noted, “I was on eggshells for the first few days purely ‘cause I didn’t know that I could act like myself” (Table S14). Prior longitudinal research showed that greater support of autonomy provided by teachers correlates with reduced depression and anxiety in teens [47]. Importantly, with freedom and autonomy comes responsibility. Adolescents had to bring their artistic visions to life. The experience of grasping the reins to carry out a mission important to oneself and others can instill a sense of purpose. Prior research shows that having a sense of purpose is critical to well-being [48] and a vital component for young people with depression to achieve greater life satisfaction, secure adult roles, develop skills, and attain an enriched identity as they emerge into adulthood [49].

Evidence from the parent data, such as their observations of an *Empowered perspective*, suggests that adolescents’ sense of responsibility translated to a new ability to take charge of things, including advocating for themselves in the face of hardships, pursuing new avenues of treatment for their mental health, and helping others. Furthermore, the themes “Enthusiasm through deep engagement” and “Sustained Enthusiasm” captured parents’ observations of a marked shift in their children’s motivation and affect that was surprising, significant, and sustained. In the six-month interviews for both adolescents and parents, vivid recollections of how adolescents navigated their camp experience —from choosing to show up each day to engaging in the creative process and completing art projects—served as tangible evidence for taking on responsibility, deep engagement, and growth.

*Discovering the uniqueness of self and others.* During the adolescent period, young people are forming their identities in critical ways [50, 51]. Adolescents with depression experience low self-esteem, holding persistently negative self-views [52, 53], even in the context of praise from others [54]. Prior research suggests a positive relationship between arts engagement and self-esteem and improved self-concept in youth [55, 56]. Creativity Camp allowed adolescents to express themselves freely and to “try on” new ways of being and seeing themselves. As one adolescent realized, in this environment, they could “be weird” (Table S14). In addition to the open environment, the curriculum’s emphasis on authentic creation through self-inquiry in the creative processes created way for adolescents to see new aspects of themselves, including a new appreciation of their own creativity. As one adolescent expressed, creativity is “tied to my internal understanding of myself and also how I try to understand the world.” (Table S14). Our findings add to prior work showing links between creativity and identity formation in adolescents [57].

The link between self and creativity continued to develop after camp. As captured in the “Expanded View of Creativity” theme, adolescents’ reflections revealed their appreciation of their own creativity. Even those who did not consider themselves as “artistic” began to see themselves as creative beings. One adolescent’s reflection on their artwork captures an important realization: “I still have the galaxy painting that we did, and I was like, ‘this actually turned out really well.’ It made me feel better about my abilities. And it made me realize I can do things.” (Table S15). By learning to navigate the world as artists, adolescents appreciated new aspects of themselves and how they fit in with others, showing evidence of personal growth in the face of depression. Witnessing the proof of their own capabilities empowered adolescents to move forward with the energy and confidence they gained from the camp experience.

Prior research also noted the importance of recognizing and appreciating the uniqueness of oneself and others. For example, a team in Colombia conducted a qualitative study on art workshops for adolescents and young adults and extracted the themes “Facilitating social support and relationships” and “Contributing to the identity of young people” from interviews with participants [58]. These themes highlight how being surrounded by youth with shared interests created a sense of community and belonging where participants did not feel “out of place”. Additionally, engaging in creative activities in the company of other young artists allowed participants to view themselves with confidence and develop an artistic identity. Another team of researchers evaluated the feasibility and impact of a two-week arts camp for high school students who survived a mass shooting [16]. The majority of participants in the study agreed or strongly agreed with the statement “Engaging in creative arts gives me a deeper understanding of myself and others.”

*Integration: Personal growth through navigating the world as an artist.* The framework of Creativity Camp, which invites adolescents to develop as artists, provides an accessible and engaging entry point for adolescents and a suitable environment to support their growth. While personal needs varied, centering the program around arts activities while allowing ample freedom to engage meaningfully and in an individualized manner with the various elements offered contributed to a sense of autonomy and structured support. Daily opportunities for arts engagement, interactions with professional artists, and opportunities to showcase their artwork with their community, encouraged adolescents to “*think like an artist*.” Arts educators have identified “studio thinking” habits of the mind that help individuals develop as creative artists [59]. Our findings suggest that engaging in the process of developing as an artist may also be helpful for mental health. This idea aligns with other recently developing ideas about arts interventions for mental health, which also emphasize the importance of deep engagement, opportunities to build social connections, autonomy, playfulness, a sense of purpose, and new and improved self-views [60–62]. The creative process is often filled with ambiguity; facing self-doubts, judgments, and fear of failure while carrying out a vision is part and parcel of any artist’s practice’s (concretely realized) creative endeavor. The practice of fostering the habits of thought and behavior that artists need to ensure their success may also help adolescents with depression, offering a potent way to shift out of negative “stuck” views and to help young people to navigate the challenges of their worlds.

## Strengths and limitations

*Strengths.* The convergent methodology adopted herein of analyzing data from several varied sources taken at multiple time points provided unique insights into the mechanisms of the Creativity Camp, with each data source complementing and reinforcing the others. For example, while observation notes captured overt behaviors and spoken interactions, they provided limited access to the reflective or introspective changes revealed during individual interviews. Conversely, adolescents may not recall or articulate specific actions during interviews, whereas observation notes documented the detailed steps they took as they engaged in creative activities. This dynamic is evident in the adolescent data, particularly in core category 2: *Exploring playfulness and responsibility*. Parent interviews offered a broader perspective, situating the adolescents’ experiences within their extended socioemotional development and beyond the immediate camp environment. This convergent approach allowed the each of the data sources to counterbalance and compensate for the limitations inherent to the other data sources, resulting in a more comprehensive understanding of the camp’s impact. Another strength of this study is the large quantity of data analyzed using the CGT method. Recent research on sufficient sample sizes for qualitative data suggests that 20–30 interviews are typically needed for Grounded Theory methods to achieve theoretical saturation [31, 63]. The sample size of the Creativity Camp study well surpasses this guideline, further underscoring the rigor and robustness of our research.

*Limitations.* First, the sample primarily represented a metropolitan population, which may not fully capture the experiences of adolescents from rural areas. Additionally, despite significant efforts made by the study team to recruit a diverse sample of adolescents, there was an overrepresentation of White and non-Hispanic youth (63%), further limiting generalizability. Second, while interviewees adhered to the study protocols, some individual responses warranted further exploration, potentially introducing information bias that favored finding supportive evidence for the beneficial effects of arts engagement. Third, although the study included multiple time points, the qualitative methods were not optimized for longitudinal analysis [64], precluding direct examination of change over time. Finally, similar to other qualitative studies, the research team’s epistemologies may have influenced the study’s interpretation and findings, despite ongoing reflection on their perspectives and positionality. In this study, the influence of the Arts in Health scholar—who emphasized bringing the artist’s perspective—was carefully considered throughout to ensure that this perspective did not predetermine the way the data was analyzed; this potential limitation was also deliberately countered by the research team members’ varied disciplinary and sociocultural experiences.

## Conclusion and clinical implications

Following our prior report showing that Creativity Camp was associated with improvements in mental health and well-being in adolescents with depression [17], the current report provides new insights into *how* the intervention works. Our findings show that adolescents developed new skills and perspectives for navigating various life challenges. They encountered the excitement and challenges of new experiences and the discomfort accompanying growth. They engaged in playful exploration while embracing the responsibilities of being an artist. They discovered their own uniqueness and creative potential. This broadening of views had a lasting impact– in the weeks and months after the camp– with a confirmed sense of their capability, appreciation of a change in their thinking and feeling, and the importance of creativity in their everyday lives. The findings suggest that Creativity Camp’s multifaceted approach, which integrated creative exploration and encouraged “thinking as an artist,” fostered significant personal growth among adolescents. This understanding may be useful in future work geared towards refining and tailoring arts interventions for adolescents with depression.

## Electronic supplementary material

Below is the link to the electronic supplementary material.

Supplementary Material 1

## Data Availability

No datasets were generated or analysed during the current study.

## References

[1] Wang S, Chen L, Ran H, Che Y, Fang D, Sun H, et al. Depression and anxiety among children and adolescents pre and post COVID-19: a comparative meta-analysis. Front Psychiatry. 2022;13:917552. 35990058 10.3389/fpsyt.2022.917552 PMC9381924

[2] World Health Organization. Mental health of adolescents [Internet]. World Health Organization. 2024 [cited 2025 Jan 27]. Available from: https://www.who.int/news-room/fact-sheets/detail/adolescent-mental-health

[3] March J, Silva S, Vitiello B. The treatment for adolescents with depression study (TADS): methods and message at 12 weeks. J Am Acad Child Adolesc Psychiatry. 2006;45:1393–403. 17135984 10.1097/01.chi.0000237709.35637.c0

[4] Weisz JR, Kuppens S, Ng MY, Vaughn-Coaxum RA, Ugueto AM, Eckshtain D, et al. Are psychotherapies for young people growing stronger? Tracking trends over time for youth anxiety, depression, attention-deficit/hyperactivity disorder, and conduct problems. Perspect Psychol Sci. 2019;14:216–37. 30571478 10.1177/1745691618805436

[5] Health Resources and Service Administration. Health workforce research [Internet]. Health Resources and Service Administration. 2024 [cited 2025 Jan 27]. Available from: https://bhw.hrsa.gov/data-research/review-health-workforce-research

[6] Abbing A, Baars EW, de Sonneville L, Ponstein AS, Swaab H. The effectiveness of Art therapy for anxiety in adult women: a randomized controlled trial. Front Psychol. 2019;10:436010. 10.3389/fpsyg.2019.01203 PMC6549595 31191400

[7] Dalli ÖE, Bozkurt C, Yildirim Y. The effectiveness of music interventions on stress response in intensive care patients: a systematic review and meta-analysis. J Clin Nurs. 2023;32:2827–45. 35668626 10.1111/jocn.16401

[8] Tang S, Xiang M, Cheung T, Xiang Y-T. Mental health and its correlates among children and adolescents during COVID-19 school closure: the importance of parent-child discussion. J Affect Disord. 2021;279:353–60. 33099049 10.1016/j.jad.2020.10.016 PMC7550131

[9] Dingle GA, Williams E, Jetten J, Welch J. Choir singing and creative writing enhance emotion regulation in adults with chronic mental health conditions. Br J Clin Psychol. 2017;56:443–57. 28722166 10.1111/bjc.12149

[10] Rodriguez AK, Akram S, Colverson AJ, Hack G, Golden TL, Sonke J. Arts engagement as a health behavior: An opportunity to address mental health inequities. Community Health Equity Research & Policy [Internet]. 2023 [cited 2025 Jan 27]; Available from: https://journals.sagepub.com/doi/10.1177/2752535X231175072 10.1177/2752535X231175072 PMC11409561 37196338

[11] Fancourt D, Perkins R, Ascenso S, Carvalho LA, Steptoe A, Williamon A. Effects of group drumming interventions on anxiety, depression, social resilience and inflammatory immune response among mental health service users. PLoS One. 2016;11:e0151136. 26974430 10.1371/journal.pone.0151136 PMC4790847

[12] Bungay H, Vella-Burrows T. The effects of participating in creative activities on the health and well-being of children and young people: A rapid review of the literature. Perspect Public Health. 2013;133:44–52. 23308007 10.1177/1757913912466946

[13] Zarobe L, Bungay H. The role of arts activities in developing resilience and mental wellbeing in children and young people a rapid review of the literature. Perspect Public Health. 2017;137:337–47. 28613107 10.1177/1757913917712283

[14] Osborn TL, Ndetei DM, Sacco PL, Mutiso V, Sommer D. An arts-literacy intervention for adolescent depression and anxiety symptoms: outcomes of a randomised controlled trial of pre-texts with Kenyan adolescents. EClinicalMedicine. 2023;66:102288. 38192586 10.1016/j.eclinm.2023.102288 PMC10772152

[15] Blomdahl C, Goulias A. Art therapy for adolescents with depression: feasibility and acceptability study in child and adolescent psychiatry. Art Ther (Alex). 2024;1–10.

[16] Hylton E, Malley A, Ironson G. Improvements in adolescent mental health and positive affect using creative arts therapy after a school shooting: a pilot study. Arts Psychother. 2019;65:101586.

[17] Cullen KR, DiMaggio-Potter ME, Klimes-Dougan B, Bernstein GA, Koutstaal W, Reigstad K et al. The impact of a Creativity Camp intervention on depression and well-being in adolescents. Child Psychiatry Hum Dev [Internet]. 2024 [cited 2024 Oct 21]; Available from: https://pubmed.ncbi.nlm.nih.gov/39412694/10.1007/s10578-024-01766-3 PMC13437562 39412694

[18] Busetto L, Wick W, Gumbinger C. How to use and assess qualitative research methods. Neurol Res Pract. 2020;2:14. 33324920 10.1186/s42466-020-00059-z PMC7650082

[19] Im D, Pyo J, Lee H, Jung H, Ock M. Qualitative research in healthcare: data analysis. J Prev Med Public Health. 2023;56:100–10. 37055353 10.3961/jpmph.22.471 PMC10111102

[20] Renjith V, Yesodharan R, Noronha JA, Ladd E, George A. Qualitative methods in health care research. Int J Prev Med. 2021;12:20. 34084317 10.4103/ijpvm.IJPVM_321_19 PMC8106287

[21] Anderson-Butcher D, Newman TJ, Amorose AJ, Okamoto K, Bates S, Volek. Exploring the influence of program staff and parental support on changes in physical health outcomes of vulnerable youth participating in a sport-based positive youth development summer camp. J Sport Behav. 2019;42:391–414.

[22] Thurber CA, Scanlin MM, Scheuler L, Henderson KA. Youth development outcomes of the camp experience: evidence for multidimensional growth. J Youth Adolesc. 2006;36:241–54. 27519024 10.1007/s10964-006-9142-6

[23] National Academies of Sciences, Engineering, and, Medicine, Division of Behavioral and Social Sciences and, Education. In: Hutton R, Sepúlveda M-J, editors. Board on children, youth, and families, committee on summertime experiences and child and adolescent education, health, and safety. Shaping summertime experiences: opportunities to promote healthy development and well-being for children and youth. Washington (DC): National Academies Press (US); 2019. 31940162

[24] Garside J, Bailey R, Tyas M, Ormrod G, Stone G, Topping A, et al. Developing a culture of publication: a joint enterprise writing retreat. J Appl Res High Educ. 2015;7:429–42.

[25] Tong A, Sainsbury P, Craig J. Consolidated criteria for reporting qualitative research (COREQ): A 32-item checklist for interviews and focus groups. Int J Qual Health Care. 2007;19:349–57. 17872937 10.1093/intqhc/mzm042

[26] Charmaz K. Constructing grounded theory. SAGE; 2014.

[27] Charmaz K, Belgrave LL. Qualitative interviewing and grounded theory analysis. In: Gubrium JF, Holstein JA, Marvasti AB, McKinney KD, editors. The SAGE handbook of interview research: the complexity of the craft. SAGE Publications, Inc.; 2012. pp. 675–94.

[28] Strauss AL. Qualitative analysis for social scientists. Cambridge University Press; 1987.

[29] Chinh B, Zade H, Ganji A, Aragon C. Ways of qualitative coding: a case study of four strategies for resolving disagreements. 2019. pp. 1–6.

[30] Greene M. On the inside looking in: Methodological insights and challenges in conducting qualitative insider research. The Qualitative Report [Internet]. 2014; Available from: https://nsuworks.nova.edu/tqr/vol19/iss29/3/

[31] Wutich A, Beresford M, Russell Bernard H. Sample sizes for 10 types of qualitative data analysis: an integrative review, empirical guidance, and next steps. International Journal of Qualitative Methods [Internet]. 2024 [cited 2025 Jan 28]; Available from: https://journals.sagepub.com/doi/10.1177/16094069241296206

[32] Flick U. Doing triangulation and mixed methods. London, England: SAGE; 2018.

[33] Vivek R, Nanthagopan Y, Piriyatharshan S. Beyond methods: theoretical underpinnings of triangulation in qualitative and multi-method studies. SEEU Rev. 2023;18:105–22.

[34] Birks M, Mills J, Francis K, Chapman Y. A thousand words paint a picture: the use of storyline in grounded theory research. J Res Nurs. 2009;14:405–17.

[35] Danitz SB, Orsillo SM, Beard C, Björgvinsson T. The relationship between personal growth and psychological functioning in individuals treated in a partial hospital setting. J Clin Psychol. 2018;74:1759–74. 29696645 10.1002/jclp.22627

[36] Thoen MA, Robitschek C. Intentional growth training: developing an intervention to increase personal growth initiative: intentional growth training. Appl Psychol Health Well Being. 2013;5:149–70. 23292959 10.1111/aphw.12001

[37] Blackie LER, Jayawickreme E, Forgeard MJC, Jayawickreme N. The protective function of personal growth initiative among a genocide-affected population in Rwanda. Psychol Trauma. 2015;7:333–9. 26147518 10.1037/tra0000010

[38] Mansueto G, Cosci F. Well-being therapy in depressive disorders. Adv Exp Med Biol. 2021;1305:351–74. 33834409 10.1007/978-981-33-6044-0_19

[39] Loijen A, Vrijsen JN, Egger JIM, Becker ES, Rinck M. Biased approach-avoidance tendencies in psychopathology: a systematic review of their assessment and modification. Clin Psychol Rev. 2020;77:101825. 32143108 10.1016/j.cpr.2020.101825

[40] Luking KR, Pagliaccio D, Luby JL, Barch DM. Child gain approach and loss avoidance behavior: relationships with depression risk, negative mood, and anhedonia. J Am Acad Child Adolesc Psychiatry. 2015;54:643–51. 26210333 10.1016/j.jaac.2015.05.010 PMC4810675

[41] Steiner S, Durk M, Marco W. Creative Art therapies in the treatment of adolescents with eating disorders: an integrative review and thematic network analysis. Arts Psychother. 2024;91:102228.

[42] Gotlib IH, Joormann J. Cognition and depression: current status and future directions. Annu Rev Clin Psychol. 2010;6:285–312. 20192795 10.1146/annurev.clinpsy.121208.131305 PMC2845726

[43] Watkins ER, Roberts H. Reflecting on rumination: consequences, causes, mechanisms and treatment of rumination. Behav Res Ther. 2020;127:103573. 32087393 10.1016/j.brat.2020.103573

[44] Ginsburg KR, American Academy of Pediatrics Committee on Communications, American Academy of Pediatrics Committee on Psychosocial Aspects of Child and Family Health. The importance of play in promoting healthy child development and maintaining strong parent-child bonds. Pediatrics. 2007;119:182–91. 17200287 10.1542/peds.2006-2697

[45] Nijhof SL, Vinkers CH, van Geelen SM, Duijff SN, Achterberg EJM, van der Net J, et al. Healthy play, better coping: the importance of play for the development of children in health and disease. Neurosci Biobehav Rev. 2018;95:421–9. 30273634 10.1016/j.neubiorev.2018.09.024

[46] Gray P. The decline of play and the rise of psychopathology in children and adolescents. Am J Play. 2011;4:443–63.

[47] Yu C, Li X, Wang S, Zhang W. Teacher autonomy support reduces adolescent anxiety and depression: an 18-month longitudinal study. J Adolesc. 2016;49:115–23. 27042976 10.1016/j.adolescence.2016.03.001

[48] McKnight PE, Kashdan TB. Purpose in life as a system that creates and sustains health and well-being: an integrative, testable theory. Rev Gen Psychol. 2009;13:242–51.

[49] Grob R, Schlesinger M, Wise M, Pandhi N. Stumbling into adulthood: learning from depression while growing up. Qual Health Res. 2020;30:1392–408. 32364433 10.1177/1049732320914579 PMC8061165

[50] Branje S, de Moor EL, Spitzer J, Becht AI. Dynamics of identity development in adolescence: a decade in review. J Res Adolesc. 2021;31:908–27. 34820948 10.1111/jora.12678 PMC9298910

[51] Meeus W, van de Schoot R, Keijsers L, Schwartz SJ, Branje S. On the progression and stability of adolescent identity formation: a five-wave longitudinal study in early-to-middle and middle-to-late adolescence: identity transitions. Child Dev. 2010;81:1565–81. 20840241 10.1111/j.1467-8624.2010.01492.x

[52] Hards E, Ellis J, Fisk J, Reynolds S. Negative view of the self and symptoms of depression in adolescents. J Affect Disord. 2020;262:143–8. 31733458 10.1016/j.jad.2019.11.012

[53] Thai M, Başgöze Z, Westlund Schreiner M, Roediger DJ, Falke CA, Mueller BA, et al. A multi-modal assessment of self-knowledge in adolescents with non-suicidal self-injury: a research domains criteria (RDoC) study. Psychol Med. 2024;54:1–12. 10.1017/S0033291724001399 39246282

[54] van Houtum LAEM, Wever MCM, van Schie CC, Janssen LHC, Wentholt WGM, Tollenaar MS, et al. Sticky criticism? Affective and neural responses to parental criticism and praise in adolescents with depression. Psychol Med. 2024;54:507–16. 37553965 10.1017/S0033291723002131

[55] Mak HW, Fancourt D. Arts engagement and self-esteem in children: results from a propensity score matching analysis. Ann N Y Acad Sci. 2019;1449:36–45. 30985011 10.1111/nyas.14056 PMC6767447

[56] Rowe C, Watson-Ormond R, English L, Rubesin H, Marshall A, Linton K, et al. Evaluating Art therapy to heal the effects of trauma among refugee youth: the Burma Art therapy program evaluation. Health Promot Pract. 2016;18:26–33. 26933006 10.1177/1524839915626413

[57] Sica LS, Ragozini G, Di Palma T, Aleni Sestito L. Creativity as identity skill? Late adolescents’ management of identity, complexity and risk-taking. J Creat Behav. 2019;53:457–71.

[58] Gómez-Restrepo C, Casasbuenas NG, Ortiz-Hernández N, Bird VJ, Acosta MPJ, Restrepo JMU, et al. Role of the arts in the life and mental health of young people that participate in artistic organizations in Colombia: a qualitative study. BMC Psychiatry. 2022;22:1–11. 36463167 10.1186/s12888-022-04396-y PMC9719131

[59] Hetland L. Studio thinking: the real benefits of visual arts education. Teachers College; 2007.

[60] Kumar V, Pavitra KS, Bhattacharya R. Creative pursuits for mental health and well-being. Indian J Psychiatry. 2024;66:S283–303. 38445283 10.4103/indianjpsychiatry.indianjpsychiatry_781_23 PMC10911317

[61] Omylinska-Thurston J, Karkou V, Parsons A, Nair K, Dubrow-Marshall L, Starkey J, et al. Arts for the blues: the development of a new evidence-based creative group psychotherapy for depression. Couns Psychother Res. 2021;21:597–607.

[62] Karkou V, Omylinska-Thurston J, Thurston S, Clark R, Perris E, Kaehne A, et al. Developing a strategy to scale up place-based arts initiatives that support mental health and wellbeing: a realist evaluation of arts for the blues. PLoS One. 2024;19:e0296178. 38165951 10.1371/journal.pone.0296178 PMC10760705

[63] Thomson SB. Sample size and grounded theory. J Adm Gov. 2010;5:45–52.

[64] Audulv Å, Hall EOC, Kneck Å, Westergren T, Fegran L, Pedersen MK, et al. Qualitative longitudinal research in health research: a method study. BMC Med Res Methodol. 2022;22:255. 36182899 10.1186/s12874-022-01732-4 PMC9526289

