# Supplementary material for: Personal growth through navigating the world as an artist: a qualitative study of the impact of creativity camp on adolescents with depression
Source: Child Adolesc Psychiatry Ment Health. 2025 Apr 2;19:38. doi: 10.1186/s13034-025-00893-6 (PMC11966796; doi:10.1186/s13034-025-00893-6)
Supplement: Supplementary file 1 — Supplementary Material 1 [file 13034_2025_893_MOESM1_ESM.docx]

**Supplementary Materials**

**Figure S1.** “The World Inside You” is a poem written by co-author Yuko Taniguchi specifically for this program. “The World Inside You” is the theme of the Creativity Camp curriculum, and the image below, designed by co-author and professional artist Peng Wu, using elements from the paintings of Suyao Tian, was used in promotional materials for the study.

**
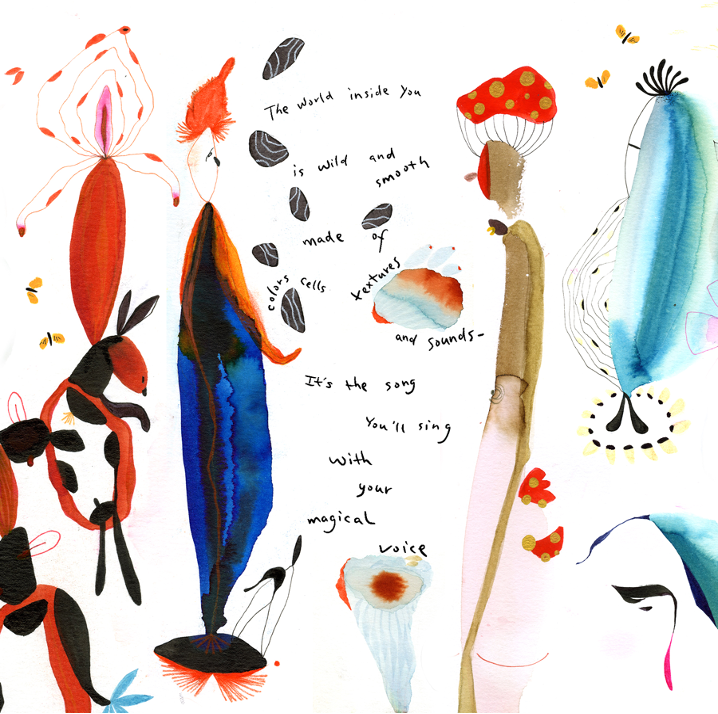
**

Watercolor artwork by Suyao Tian. Poem by Yuko Taniguchi. Composition by Peng Wu.

| **Table S1: Completed Sample Characteristics (N = 77)** | |
| --- | --- |
| **Characteristic** | *n* (%) |
| **Age, mean (SD)** | 15.1 years (1.65) |
| **Race/Ethnicity** |  |
| African American/Black | 7 (9.1) |
| Asian | 4 (5.2) |
| Native American | 1 (1.3) |
| Multiracial | 10 (13.0) |
| Non-White & Hispanic/Latinx | 1 (1.3) |
| White & Hispanic/Latinx | 5 (6.5) |
| White & Non-Hispanic/Latinx | 49 (63.6) |
| **Sex at Birth** |  |
| Female | 60 (77.9) |
| Male | 15 (19.5) |
| Prefer Not to Say | 2 (2.6) |
| **Gender Identity** |  |
| Transgender/Nonbinary | 22 (28.6) |
| Cisgender Woman | 34 (44.2) |
| Cisgender Man | 15 (19.5) |
| Other | 6 (7.8) |
| **Parent Education** |  |
| High School, No Diploma | 2 (2.6) |
| High School Graduate | 2 (2.6) |
| Some College/No Degree | 12 (15.6) |
| Associate’s Degree | 8 (10.4) |
| Bachelor's Degree | 24 (31.2) |
| Master’s Degree | 21 (27.3) |
| Professional School Degree | 3 (3.9) |
| Doctoral Degree | 5 (6.5) |
| **Parent Employment** |  |
| Working | 64 (83.1) |
| Unemployed | 2 (2.6) |
| Keeping House | 5 (6.5) |
| Student | 2 (2.6) |
| Other | 4 (5.2) |
| **Mental Health Diagnoses** |  |
| At least one Mental Health Diagnosis | 72 (93.5) |
| Depressive Disorders |  |
| Current Major Depressive Episode | 43 (55.8) |
| Major Depressive Disorder | 67 (87) |
| Bipolar Disorders |  |
| Bipolar Disorder I | 6 (7.8) |
| Bipolar Disorder II | 2 (2.6) |
| Other Specified Bipolar Disorder | 1 (1.3) |
| Anxiety Disorders |  |
| Any Anxiety Disorder | 56 (72.7) |
| Generalized Anxiety Disorder | 32 (41.6) |
| Specific Phobia | 16 (20.8) |
| Social Anxiety Disorder | 27 (35.1) |
| Panic Disorder | 17 (22.1) |
| Separation Anxiety Disorder | 12 (15.6) |
| Agoraphobia | 19 (24.7) |
| Unspecified Anxiety Disorder | 1 (1.3) |
| Trauma and Stressor-Related Disorders |  |
| Posttraumatic Stress Disorder | 10 (13.0) |
| Others |  |
| Attention Deficit Hyperactivity Disorder | 10 (13.0) |
| Eating and Feeding Disorders | 11 (14.3) |
| Tourette's/Motor/Phonic Tic Disorder | 11 (14.3) |
| Obsessive Compulsive Disorder | 7 (9.1) |

**Table S2. The Creativity Camp Curriculum: The World Inside You.** One key aspect of this curriculum is that it aims to instill the sensation that the participants are going in a direction that they have not gone before. The curriculum is designed to have the participants connect to the topics in new ways that they had not considered previously by introducing the topics with a creative twist. While participants may have their own understanding of the topics, or they may be familiar with the medium of the art project, they likely have not explored and expanded their perspectives through the specific contexts introduced in the curriculum. A second key aspect of the curriculum is that it is structured to support self-inquiry. The theme of each session intends to explore the relationship between a specific topic and the self, “me,” referring to the participants. The curriculum encourages participants to gain a new relationship between themselves and each topic, which is revealed through their creative work. The table below describes each of the sessions’ themes and activities. There were some changes to the specific activities between 2022 and 2023; these are noted below.

| **Day** | **Theme** | **Medium options** | **Questions to be explored & the overview of activities** |
| --- | --- | --- | --- |
| 1 | Multitude & me | Poetry and Collage | What makes me, me? Based on a poem, “Begin With Pieces,” by co-investigator Taniguchi, participants engage in creative writing to explore who they are. Through a series of prompts which instill holistic reflection of the whole person, they extract what they love, what follows them, and where in their body they hold darkness. These prompts connect to the participants’ key concepts that represent their lived experiences. Based on these themes they identified, they decorate 12 pieces of a wooden jigsaw puzzle with mixed media to create an abstract self-portrait. This creative process aims to have the participants recognize the multitude nature of the self and the beauty of various experiences being represented visually. |
| 2 | Chaos & me | Clay, drawing, and dance | How does chaos feel inside our minds and bodies? Participants pay attention to their internal system: heart and brain. They recognize the music inside us created by our heart that tirelessly beats and our mind with the ability to imagine. Through listening to their own heartbeats and reflecting on how they imagine, they contemplate how both our hearts and our minds help us to work through chaos instead of being swallowed by it. Participants are invited to capture their internal system through abstract line drawing (2022) and clay (2023). Then they are invited to physically experience chaos collectively through contra dance. The aim of this session is to reconsider and redefine chaos, which may be stressful, yet is an opportunity to tap into our bodies and skills to creatively navigate. |
| 3 | Nature & me | Letter to the river and nature walk | What if nature isn’t a place to visit but a space we absorb inside us? What is my connection to the sky, the sun, and the river? In this session, we explore our connection to nature creatively by first composing a letter of gratitude to the Mississippi river on compostable paper that responds to the question, What do you want the river to know? Participants journey to the river together to send their letters, and on this journey, they explore the surrounding natural world, and collect natural materials to be used in a subsequent activity (in 2022, the Community Forest activity in Session 4; in 2023, the Safe Space activity in Session 5). Participants engage in thinking creatively about breathing in and taking in the warmth of the sun as they imagine that nature and its power can stay with them. |
| 4 | Community and me | Collaborative art project | What gets created when we collaborate with others in our community? Following up on the previous nature session, participants are invited to consider questions at the macro level like what larger spaces, such as the forest, the sky, and the broader universe look like. Then they are guided to shift their perspectives to the micro level by noticing what is in each space.  2022: Community Forest. Through a multimedia collage of community forest creation, participants explore what exactly is included in a forest including nature's communication system of mycorrhizal networks. Participants are invited to use origami and their collections from the previous session’s nature walk to collaboratively create a one-of-a-kind, unique community forest.  2023: Universe. Participants are guided to paint the universe with their own unique versions of the night sky. Then, their macro view is shifted to ponder the micro level prompt: “What is missing from our universe? What are the things you wish you could see/have?” Each participant paints an individual canvas, which are all later joined together, to create a community mural that fills in the world’s missing pieces through art.  Overall, this session aims for the participants to shift their perspectives from larger concepts to details to notice their environment and explore the collaborative creative process to discover and witness what gets created when many ideas are combined. |
| 5 | Space & me | Graphic design, 3D printing and mixed media collage | How do different environments influence us? When and where do we feel comfortable? Fom designing one-of-a-kind chairs that represent their creativity and comfort (2022) to creating a safe space mixed-media sculpture (2023), this session aims for the participants to become aware of key designs that are connected to comfort, safety, and wellbeing and express it through artwork.  2022: Designing comfort. Participants explore various types of space and designs in the built environment all over the world to collect their responses to each space. In addition to paying attention to technical details like size, shape, and color of the furniture and design of each space, they also collect how they feel about each space. Then they are invited to design an external object that represents comfort and safety, which is then 3D printed.  2023: Safe-space collage. Participants are invited to use their collections from the nature walk to incorporate them into their safe space mixed-media collage sculpture. |
| 6 and 7 | The World Inside and Me | Clay or abstract painting | What does the inside of your mind look like? What ideas, stories, thoughts, and emotions are inside you? What color are they? And how do they move? Participants are invited to make their internal mind into a concrete physical object.  2022: Clay boxes. Participants are invited to create a black clay box with a lid with instructions to keep the external surface plain, in which they can place a 3D-printed scaled-down version of their brain (created from the neuroimaging data that was collected in 2022 only). But inside the box - on the inside clay walls and the inside top of the box - they are encouraged to make their imagination visible, showing the various imaginations they hold inside them through their artwork.  2023: Abstract painting. What are you most passionate about? What does the inside of your mind look like? What ideas, stories, thoughts, and emotions are inside you? What color are they? And how do they move? Participants are invited to “go big and be bold” through abstract painting to express their inner selves and passions through colors on a large canvas with instructions to keep the expression abstract, avoiding concrete figures.  Overall, this session challenges participants to consider their imagination as if it is another world inside and communicate their vision with an external audience. |
| 8 | Reflection and Sharing | Artist Interview | What have you discovered thus far? What do you notice from everything that you have created during this camp? How will you carry your creative self into the future? In this session, participants reflect on what they have created. They are invited to learn from their own creations and share what they have learned about their own creativity. The interviewer and participant examined a few of the art projects the participant created during the camp in this interview and the participant shared their thoughts on the final product. |

**Table S3.** Schedule Structure - Summer 2022

| 12PM-12:45PM | Campers arrive  Lunch* |
| --- | --- |
| 12:45-1:15 PM | Creativity warm-up exercises |
| 1:15-2:30 PM | First round of creative arts activities |
| 2:30-2:45 PM | Snack & break |
| 2:45-4PM | Second round of activities & wrap up |
| 4-4:15PM | Campers are picked up to return home |

**Table S4.** Schedule Structure - Summer 2023

| 10AM-10:45PM | Campers arrive  Art exercises introducing theme of the day |
| --- | --- |
| 10:45-12:00 | First round of creative arts activities |
| 12:00-12:45 | Lunch* |
| 12:45-2PM | Second round of activities & wrap up |
| 2:00-2:15 | Campers are picked up to return home |

*For the lunches, to enhance the camp’s novel experiential components, including new conceptual and sensory-perceptual experiences, food was ordered from culturally diverse local restaurants, featuring Japanese, Thai, Indian, Cambodian, Italian, Vietnamese, Middle Eastern, and American cuisines.

**S5. Protocol for Taking Observation Notes - Instructions for Near-Peer Facilitators**

Ethnography is a way to learn more about individuals, groups, and communities in their own socio-cultural environment using qualitative methods such as interviewing, photovoice, participant observation, etc.

After each session, you will develop ethnographic notes that describe and analyze your experience as a participant observer. Your role as a participant observer means you will create data to help us better understand the social “life” occurring before you.

During the sessions, you will briefly jot down your observations in a small notebook; after the sessions, you will expand these notes to create more in-depth field notes. This approach helps balance the need to capture important phenomena while you are still “in the moment” (so you can remember them later by your brief notes), with the need to avoid disrupting the flow of the sessions, and minimizing the risk of adolescents feeling like they are “under the microscope” due to the observation note-taking process.

**Our overall inquiry**

How does engagement in creative activities positively impact adolescents?

**Our research question(s)**

1. Does deep engagement in creative activities lead to more flexible thinking/brain flexibility, positive views of self and the world, and a better mood?
2. Since we are studying flexible thinking, how do adolescents hold multiple views of themselves in which the negative does not dominate?

PROTOCOL:

Observer’s Name: Date of observation:

1. During the day, use your field notebook to take ethnographic field **jottings** that will remind you what to write during the **field notes** step.
2. After the conclusion of that day’s camp activities, you will create ethnographic field notes. You have the option of typing these or creating an audio note. (I would prefer audio notes.) As you read your jottings, use the following questions to guide your field note creation:

**Part I: Field Site Analysis Guiding Questions^^[[1]](#footnote-1)^^**

- 1. What was the overall “feel” of the day?
  2. Thinking of a **specific encounter/incident/or specific encounters/incidents that stood out from the day…**
     1. …Who were the participants engaged in this action? Describe in detail -- **PAINT A PICTURE IN MY MIND.**
     2. What was happening? What are the adolescents trying to do?
     3. **When looking at these encounte**rs, **WHERE AND HOW YOU SEE?**
        1. **Engagement:** How are adolescents showing interest? How do they show enthusiasm? What does it look like when they are working hard at something? How are they elaborating? How do they add new ideas to something? How do they share their work with others? How do they talk to others in general? OR Are adolescents withdrawing? If so, how? Are they not showing enthusiasm? If so, how? Are they not participating in the activities? If so, how? Are they refusing to share? If so, how? Are they not talking to others around them? If so, how?^^[[2]](#footnote-2)^^
        2. **Enjoyment/positive mood:** Am I seeing adolescents smiling? laughing? talking? sharing stories? positive emotional tone of voice? What does positive content of what they say look like? How do they talk about themselves? How do they talk about others around them? About the world? Or am I seeing the opposite? Sad face/sadness or flat affect, crying, flat tone of voice, anger/irritability, pessimistic views, and talk? Negative self-talk?
        3. **Resistance:** How is resistance to activities, tasks, ideas, etc. being expressed?
        4. **Working with discomfort:** How does participants' initial reaction to difficulty or discomfort they have faced (frustration, desire to give up, expression of disappointment that things are turning out, resistance/fear of trying something new) look like?
        5. **Idea development:** How do they speak about their ideas? How do they describe where they come from? How do they describe how they arrive at them? How did it influence them?
        6. **Insight:** How are adolescents expressing feelings of surprise about new ideas? Excitement/interest to elaborate?
        7. **Expression of curiosity:** How is curiosity expressed?
        8. **Creative exchange:** Are adolescents showing idea sharing between themselves and others? Do they encourage or compliment each other? If so, how?
        9. **Social exchange:** How does a sense of connection between adolescents or between teens and staff look like? Are they building trust? Sharing personal aspects? Looking forward to shared time together? Are they sad at parting from each other when camp finishes? Sadness at parting?
     4. How, exactly, do they do this? What specific means/or strategies do they use? For example, HOW does “expressing feelings of surprise” look like?
     5. How do adolescents talk about, characterize, and understand what is going on? For example, HOW does the adolescent ask questions about specific activities/tasks?
     6. How successful are they at accomplishing this?

REMEMBER: The “HOW” IS TELLING PAINTING A PICTURE IN MY MIND. YOU WILL NEED TO ENGAGE IN A LOT OF DESCRIPTION IN YOUR WRITING/AUDIO NOTE TO SHOW ME THE “HOW.”

**Part II: Reflective and Analytical Synthesis Guiding Questions -- Ethnographer as Researcher^^[[3]](#footnote-3)^^**

- 1. As a whole, what do I see going on here this day? Why?
  2. What did I learn from this day? What incidents/encounters stood out?
  3. How did it/they make me feel?
  4. What surprised me? What didn’t? Why?
  5. What was I expecting to see today that I did not see before? What was missing or not happening today?
  6. What issues, problems, and concerns are affecting/impacting the campers I observe? Why?
  7. What aspects of this day create more questions? What are these questions?
  8. Thinking back on our main inquiry and our research questions… How do the day's events answer/complicate/challenge our inquiry and/or questions? Why is that?

**S6. Final Camp Day Participant Interview Questions**

QUESTIONS:

[FOR EACH CREATIVE PRODUCT, ASK QUESTIONS 1-9, BY SHOWING THE PIECES]

1. If you could give this work a title, what would it be? Could you tell me more about why you titled it this way?
2. What do you like about this piece?
3. What else can you tell me about this piece?
4. What emotions were happening when you made this piece? Why do you think you experienced these emotions?
5. Now that the piece is complete, how do you feel when you look at it now? Why is that?
6. What do you want viewers to feel when they look at this piece? Why is that?
7. Imagine seeing this piece you created five years from now. What do you think this work will remind you of?
8. If someone was to ask you about this piece ten years from now, what would you want them to understand about you back then? Why is that?
9. Is there anything that you learned about yourself from making this piece that you weren't aware of? What do you know about yourself now that you did not know before?

[AFTER YOU HAVE FINISHED ASKING THE QUESTIONS FOR EACH OF THE ARTWORKS, ASK THE FOLLOWING QUESTIONS]

1. What other types of work would you want to make in the future?
2. How did these projects inspire you to make more art?
3. How did you decide to participate in the creativity camp?
4. You have attended this camp every day. Was it difficult to show up everyday? Or were you excited? Tell us your experience.
5. On Day 1, you learned about the definition of curiosity: sensation of liking something & feeling weird. What felt weird to you during the camp? What brought out your weirdness? (This does not have to be art-related experiences. It can be anything from the camp.)
6. Was there a moment during the camp when you had to step out of your comfort zone? Describe what happened.

**S7. Six-Month Participant Interview Questions**

QUESTIONS:

1. Thinking back to the creativity camp last summer, could you tell me three things that you fondly remember from the camp? These three things don’t need to be about class or the artwork you created. Think about everything you have done, from having lunch, talking to peers, and making art. What comes to your mind?
2. Probe: Why do you think these things were memorable to you? How did they impact you? How did you feel during these experiences?
3. Now, think of three things that felt challenging to you during the camp. Again these three things don’t need to be about class or the artwork you created. What comes to your mind?
4. Probe: Why do you think you experienced these emotions? How did you navigate these challenges? (For example, did you talk to the undergraduate student volunteers? Did you talk to your family?) Why do you think these things were challenging/uncomfortable/difficult?
5. What did you discover about your creativity during the camp? How did this discovery influence you after the camp?
6. In Creativity Camp, we encouraged the artist mindset. Taking on this mindset means being curious, flexible, and boldly expressing yourself in a way that is authentic (e.g., our "weirdness" activity during Camp). Since Creativity Camp, have you used this artist's mindset? More specifically, can you recall being curious, flexible, weird, or bold, or have you been playful or shared your creations with others since Camp? If so, can you provide examples?**^2^**
7. What are some of the things you have been enjoying and participating in since the Creativity Camp? Have you noticed anything about yourself or your activities either during or after school in the following categories? (It is okay if some of these responses overlap).**^2^**
8. Clubs or activities during school, after school, or on the weekends
9. Physical activities/activities that involve movement
10. Can be structured, e.g., sports, dance groups
11. Can be unstructured/informal, e.g., walking, bike riding
12. Social engagements with friends or family
13. Can be structured, e.g., clubs
14. Can be unstructured/informal, e.g., hanging out with classmates, spending time with family
15. Creative activities
16. Can be structured, e.g., art classes, vocal lessons
17. Can be unstructured/informal, e.g., knitting, scrapbooking
18. Outdoor activities
19. Can be structured, e.g., gardening club, outdoor sport
20. Can be unstructured/informal, e.g., hiking, tending to plants
21. For the clubs and activities previously discussed, do you feel like your participation has decreased, increased, or stayed the same since before participating in the Creativity Camp?
22. In the future, you might use your creativity to support a program like the Creativity Camp. If you could develop your own program for teens, what kind of class would you like to develop? Feel free to think out loud as you think about a class you would lead.

[ASK THE FOLLOWING QUESTION BY SHOWING THE PHOTO OF THE SELF-PORTRAIT PIECE THAT THE PARTICIPANT CREATED]

1. Let’s talk about the self-portrait you created. How do you feel about your work today? What do you notice about the work that you didn’t notice before?
2. [GIVE A BLANK PUZZLE PIECE]* Using this blank puzzle piece, if you could add a piece today, how might you decorate this piece?**^1^**
3. Your work will be displayed at Weisman Art Museum in Spring. Could give this work a title? Could you tell me more about why you titled it this way?**^1^**
4. How would you like your name to be displayed? (Example: First name, middle initial, and last name; First and last name; Just first name; Or other creative option.)**^1^**

WRAP-UP:

- Is there anything else you would like to share or would like us to know?
- Are there any questions I did not ask that I should ask the next person I interview?
- Follow-up: So how would you answer that question?

**^1^Questions only asked during Summer 2022 follow-up visits**

**^2^Questions only asked during Summer 2023 follow-up visits**

**S8. Immediately Post-Camp Parent Interview Questions**

1. How did you learn about the creativity camp study, and why did (Child’s Name) decide to participate in this opportunity?
2. What kind of experiences from the Creativity Camp did (Child's Name) talk about at home?
   1. If they talked about their experiences…

- What did they talk about? How did they talk about it?
- What was your reaction?
- What felt new to you or what surprised you from these conversations?
- What were you expecting from the art work?

1. Did (Child’s Name) talk about the people they met during the camp? If yes, what was (Child’s Name)’s impression of the community of this Creativity Camp?
2. Have you noticed any new artistic or creative activities (Child's Name) has done at home?
3. Did (Child's Name) share or describe some of the artwork they created?
   1. If yes,

- What kind of conversation(s) did you have?
- Did (Child’s Name) explain what they were trying to express?
- Did (Child’s Name) explain what they liked?
- What was your reaction to the artwork (Child’s Name) described?
- What did you notice or learn about your child from this conversation through the artwork?
  1. If no,
- No follow-up conversation is necessary.

1. Have you noticed any changes in your child taking initiative to be more social or more physically active? Could you give me an example of this?
2. Is there anything else you would like to share with us?

**S9. Six-Month Parent Interview Questions**

QUESTIONS:

1. Does (Child’s name) mention the creativity camp experiences since it was completed? If so, what was the occasion, and how did they talk about it? Please provide an example.
2. Has (Child’s name) been engaged in any new artistic or creative activities since the camp?
3. What changes (if any) do you see in (Child’s name) and how they have been living their life, that may have come from the camp? Have you noticed any changes in your child in the following areas? If so, please provide specific examples.

- Outlook in life
- Relationships (family and friends)
- Taking initiative in social life
- Taking initiative in being more physically active
- Willingness to try new things

1. From your perspective, what do you think (Child’s name) discovered about themselves during the camp?
2. Do you think this camp had an impact on (Child’s name) from your perspective? Have you noticed any instances where (Child’s name) seemed to be applying new skills / wisdom that they had gained from the camp in their day-to-day life?

**S10. Reflexivity of the qualitative data research team.**

This section focuses on the perspective and positionality of team members who carried out the qualitative methods. The qualitative methods used in this study utilized an interpretive approach, recognizing the existence of multiple realities. For navigating the complexities of research across different cultures and contexts, the transparency of the researcher's perspective or position is essential to demonstrate the rigor of the study and credible findings (Louis, 1992; Ritchie et al., 2009). Opie and Sikes (2004) discussed the importance of qualitative researchers critically reflecting on their philosophical and paradigmatic stance, as their assumptions inevitably shape their research approach. For this purpose, we aim to acknowledge and transparently communicate the backgrounds of the participatory observation note takers and interviewers involved in qualitative data collection, as well as provide a more in-depth description of the positionality of the team members who participated in data analysis.

**Participatory Observation Note Takers.**  These research members, also referred to as near-peers, include undergraduate students and post-baccalaureate scholars who observed adolescent participants and took participatory observation notes. Their academic focus is within STEM disciplines, including Psychology, Health Sciences, Biology, and Pre-Medicine. These students were either brand new to research or had minimal prior research experiences. All students were interested in pursuing professional careers related to health sciences.

### Interviewers. The interviews were conducted by an interdisciplinary team. A total of 29 interviewers participated in the study. Among them, 21 identified as women, 7 as men, and 1 as non-binary. Their academic credentials included 9 undergraduate students, 10 interviewers with a Bachelor of Science degree, 2 interviewers with a Bachelor of Science in Health Sciences, 1 medical student, 2 psychiatrists, 2 psychologists, and 2 professional artists. Their perspectives were informed by clinical research, patient care, and creative practice.

**Qualitative data analysis team.**  The lead researcher (first author, YT) oversaw the entire qualitative data analysis process, from supervising the initial codebook development to coding throughout the CGT processes. She identifies as a woman and is an Arts in Health scholar with expertise in qualitative analysis. She holds a Master of Fine Arts (MFA) degree and is a novelist, poet, multimedia artist, and educator. Her perspective is shaped by her artistic training, lived experiences as a 1.5-generation Japanese immigrant, and two decades of experience providing creative writing and art workshops for patients and practitioners. The second author (OC), the fourth author (SH), and the fifth author (JF) served as participating researchers and interviewers and conducted the axial and focused coding of qualitative data. They identify as women, hold Bachelor of Science degrees in Psychology, and have been trained in clinical psychology with a focus on adolescent depression and well-being. The third author (AO) conducted interviews for parents and completed the axial and focused coding of the parent qualitative data. She identifies as a woman, holds a Bachelor of Science in Health Sciences, and was trained in qualitative analysis within the fields of social science and Arts in Health. The seventh author (JB) served as a data analyst, overseeing the research team members responsible for initial and focused coding. He identifies as a male and holds a Master of Science in Mathematics with an Education Emphasis and a Master of Education in Math Education. With over a decade of experience as an educator in mathematics, statistics, and research methods, his perspective is shaped by his expertise in curriculum design and the development of learning activities.

### Research team members for initial and focused coding. Twenty undergraduate Health Sciences students conducted the initial and focused coding under the supervision of the lead researcher and the seventh author. They were enrolled in a course dedicated to qualitative analysis of creativity and its impact on mental health. They were not a part of the intervention or data collection.

**References**

Louis, M. R., & Bartunek, J. M. (1992). Insider/outsider research teams: collaboration across diverse perspectives. *Journal of Management Inquiry*, *1*(2), 101-110. <https://doi-org.ezp2.lib.umn.edu/10.1177/105649269212002> (Original work published 1992)

Ritchie, J., Zwi, A. B., Blignault, I., Bunde-Birouste, A., & Silove, D. (2009). Insider–outsider positions in health-development research: reflections for practice. *Development in Practice*, *19*(1), 106–112. <https://doi-org.ezp2.lib.umn.edu/10.1080/09614520802576526>

Opie, C. (2004). *Doing educational research: A guide to first-time researchers*. SAGE Publications Ltd, https://doi.org/10.4135/9781446280485

**Table S11. Participant Data (Interviews and Observation Notes) – Initial Coding Framework**

| **Categories** | **Initial Codes (38)** | **Definition** |
| --- | --- | --- |
| Creative Process | Purpose | Seemed to be connecting to a purpose while creating |
|  | Discovery | Became aware or/and discovered their own personal traits, personality features, styles, habits, etc. that they were not aware of before |
|  | Depth | Tapped into deep thoughts, ideas and feelings |
|  | Curiosity | Addressed various questions including existential questions about the nature of the world, of society, or of humankind. |
|  | Exploration | Practiced taking risks, trying new things |
|  | Introspective | Reflected to get in touch with the self, their ideas, their lived-experiences, memories |
|  | Whimsical | Playful way of thinking and creating |
| Emotions | Uncomfortable | Navigated discomfort |
|  | Joy | Experienced joy, fun, and enjoyment through the course activities or creative activities |
|  | Proud | Experienced feeling proud of themselves or impressed by what they did |
|  | Surprised | Experienced being surprised by their own discovery |
|  | Inspired | Experienced feeling inspired |
|  | Motivation | Experienced feeling motivated |
|  | Free | Experienced feeling liberated |
|  | Hopelessness | Expressed negativity or pessimism about the future; despair |
|  | Hopeful | Expressed being able to see beyond limitations, experienced feeling hopeful |
|  | Struggles | Understood their own and/or other's suffering |
|  | Anxiety | Experienced anxiety, nervousness, and fear |
|  | Sad | Experienced sadness |
|  | Angry | Demonstrated anger |
|  | Gratitude | Felt grateful for the creative experience |
|  | Light | Expression of delight, light mood |
|  | Heavy | Expression of heavy feeling |
| Self-view | Trust | Trusted their own abilities to get through activities or situations |
|  | Authenticity | Recognized that their expression was uniquely their own, that only they could capture their expression in ways they did |
|  | Validation | Experienced being valued or appreciated through their creative work |
|  | Compassion | Displayed compassion toward the self and/or ability to think about themselves in a compassionate way |
|  | Future Thinking | Explored what they might do or be in the future |
|  | Holistic | Demonstrated their holistic understanding of the self that is comprised of multiple elements |
|  | Rigid | Demonstrated their self and the world view as fixed and limited |
| Community | Friends | Made new connections and friendships |
|  | Safety | Expressed that they felt safe with their community |
|  | Belong | Felt they belonged to their community |
|  | Indifferent | Felt indifferent about the community and connection |
|  | Sharing | Shared their work, ideas, or feelings with others |
|  | Guarded | Kept to themselves, did not interact with others |
| Growth | Change | Expressed change in any behavior, feelings, or awareness since the camp started |
|  | Stretched | Expressed that they experienced expansion, stretch of the self |

**Table S12. Participant Data Focused Coding to Sub-category Framework**

| **Category** | | **Focused Codes (16)** | **Definition** | **Informed by Initial codes** |
| --- | --- | --- | --- | --- |
| **Creative Process** | | Depth | Tapped into deep thoughts, ideas, feelings, and/or understanding through introspection, reflection and recalling | "Introspective," "Struggled," "Sad," "Anger," "Light," "Heavy," "Change" |
|  |  | Curiosity | Addressed various questions including existential questions about the nature of the world, of society, or of humankind. | "Holistic" |
|  |  | Exploration | Practiced taking risks, trying new things, playing | "Whimsical," "Stretched" |
| **Vulner-ability** | **Mixed emotions** | Uncomfortable | Navigated discomfort |  |
|  |  | Joy | Experienced joy, fun, and enjoyment through the course activities or creative activities |  |
|  |  | Inspired | Experienced feeling inspired, liberated and open | "Proud" |
|  |  | Motivation | Experienced feeling motivated |  |
|  |  | Anxiety | Experienced anxiety, nervousness, and fear | "Rigid" |
|  | **Self-view** | Authenticity | Authenticity | "Purpose" "Discovery" |
|  |  | Validation | Experienced being valued or appreciated through their creative work | "Trust" "Compassion" |
|  |  | Future Thinking | Explored what they might do or be in the future |  |
|  | **Community** | Friends | Made new connections and friendships |  |
|  |  | Belong | Felt they belonged to their community | "Safety" |
|  |  | Indifferent | Felt indifferent about the community and connection |  |
|  |  | Sharing | Shared their work, ideas, or feelings with others |  |
|  |  | Guarded | Kept to themselves, did not interact with others |  |

**Table S13. Parent Interview Coding Framework**

| **Category** | **Initial Codes (70)** | **Code Definition** |
| --- | --- | --- |
| Context | Background | Parent described the child's past experiences and background to provide context in order to understand the meaning of current behaviors. |
|  | Thrive | Parent described what allows the child to thrive based on their past (this is often connected to what the camp offered, below). |
|  | Camp & Thrive | Parent indicated that the camp connected the participants and the ingredients that make them thrive. |
| Sharing | Share | Parent described that the child shared about the camp to them or someone. |
|  | Somewhat | Parent described that the child shared a few things from the camp to them or someone. |
|  | No share | Parent said that they don't know much about what the child did at the camp since the child did not share about the camp. |
| Camp Activities | Lunch | Parent mentioned that the participants described specific activities from the camp, indicating that these experiences were memorable, influential, and/or meaningful to the participants. |
|  | Portrait |  |
|  | Dance |  |
|  | Nature Walk |  |
|  | Safe Space |  |
|  | Abstract Painting |  |
|  | Clay |  |
|  | Origami |  |
|  | Writing |  |
|  | Galaxy Painting |  |
|  | Community Piece |  |
|  | Overall | Parent mentioned that the child enjoyed the camp overall. |
|  | Taking artwork home | Parent described enthusiasm in their child about taking their artwork home after camp. |
| Discovery | Authenticity | Parent noticed that the child became aware of their own creativity and authenticity, that their expression is specific and uniquely their own |
|  | Curious Future | Parent noticed that the child became curious about things that they may explore in the future |
|  | Interest | Parent noticed that the child discovered an area they want to do and learn more about |
|  | Past-self | Parent noticed that the child discovered about their own past through reflection |
|  | Evolving | Parent noticed that the child experienced that life/people/situation/feelings/thoughts are constantly changing |
|  | Problem Solve | Parent noticed that the child navigated a situation to solve an issue on their own |
|  | Preference | Parent noticed that the child tried various things, that they became aware of what they like and what they don't like |
| Emotional Experience | Challenged | Parent noticed that the child felt challenged or disappointed through difficult or uncomfortable situations |
|  | Compare | Parent noticed that the child compared themselves to other peers |
|  | Appreciated | Parent noticed that the child experienced being appreciated, celebrated, respected, recognized, and seen during the camp. |
|  | Engaged | Parent noticed that the child was engaged, energized, and/or motivated during the camp due to having a purpose and/or experiencing encounters and projects that were meaningful to them |
|  | Enjoyed | Parent noticed that the child experienced enjoyment, joy, and/or fun |
|  | Liked | Parent heard about the child liking the camp, certain activities, and expressing the desire to go to the camp |
|  | Surprised | Parent learned that the child experienced feeling surprised |
|  | Relaxed | Parent noticed that the child felt relaxed and peaceful at the camp |
|  | Open | Parent noted that the child expressed that they felt free, open, and liberated |
| Camp Relationship | Peers | Parent heard about impactful relationships |
|  | Staff |  |
|  | College students |  |
|  | Artists |  |
|  | Belonged | Parent noticed that the child felt comfortable in the camp community and/or felt they fit in and belonged. |
|  | Didn't belong | Parent noticed that child did not feel connected to other peers and did not have a strong social experience with other peers |
| Camp Culture | Pace (Camp Structure) | Parent commented on the structure of the camp, which maintained a busy pace and including various activities |
|  | Integrated | Parent noticed that everyone (participants, strudents, peers) participated and created together |
|  | Research | Parent described the meaningfulness of being a part of a scientific study, including signing up, being scanned, getting paid |
| Improved Behavior since the Camp | Physical Change | Parent noticed a change/increase in physical activity and health habits (e.g., more sleep, healthier diet, less technology) |
|  | Family Change | Parent described changes in family dynamics or interpersonal interactions at home or with immediate family, which may include outward behaviors, actions, and changes in routine |
|  | Peer Change | Parent perceived changes in interpersonal dynamics with peers outside the family |
|  | Outing | Parent noticed the increase of outing experience (shopping, taking a bus, etc) |
|  | Creativity Change | Participants do new or more creative activities at home or continued their creative habits |
|  | Perspective Change | Parent noticed that the child's perspective (the way they view their life) has shifted |
|  | Decrease maladaption | Parent perceived a decrease in maladaptive behavior |
| Challenged state of being since the camp | Mood-Negative | Parent noticed that the child displayed negative mood and/or dissatisfaction at home because the camp was so much fun |
|  | Sad | Parent noticed that the child displayed sadness due to the camp ending. |
|  | Mood-no change | Parents noticed that the child's mood remained the same. |
| Improved state of being since the camp | Willingness improved mood | Parent noticed that the child is willing to do/try things |
|  | Enjoyment improved mood | Parent noticed that child was having fun and enjoying activities |
|  | Mood improved | Parent noticed that the child's mood was improved |
|  | Empowered | Parent noticed that the child seems empowered, inspired; they are carrying on in life with a more energized state of being. |
|  | Confident | Parent stated that the child displayed more confidence |
| Impact on parents | Deeper understanding | Parent expressed that they gained a deeper understanding of their own child |
|  | Respect | Parent recognized and appreciated their child's creativity and showing respect as an artist |
|  | Unexpected | Parent noticed the child's action/behaviors were unexpected, different from their usual behaviors |
|  | Parent action | Parents expressed the intention to take acton or took actions as a response to the child's behavior in response to the camp |
|  | Parent curious | Parents began wondering at a new aspect of their child |
|  | Future | Parents described the positive outlook of the child's future |
|  | Hopeful | Parents felt hopeful that their child will be likely to improve because of this camp |
| Homelife | Overlook | Parents' comments or behaviors during the interview indicated that their child is not the focus of parent attention. |
|  | Stigma | Parents' comments or behaviors during the interview indicated that they hold a negative bias toward mental illness |
|  | Difficult Home | Parents' comments and behaviors during the interview indicated that the child navigates home life that may be burdensome, unhealthy, and/or unsafe. |
|  | Support | Parents' comments and behaviors during interview indicated that the child has a supportive and validating homelife and parent is very well aware of their child's mental health and well-being |

**Table S14. Adolescent Data - Representative Quotations**

To protect participant confidentiality, all names throughout have been changed to pseudonyms, and all pronouns have been changed to “they/them.”

| **Core category** | **Representative quotation *(I*: Interview; *O*: Observation)** |
| --- | --- |
| 1. Internal negotiation between novelty and discomfort | *O*: At the beginning of the dancing activity, Charlie was very opposed to touching hands. They were right behind the dance instructor who was instructing them to grab onto their hand, but they were uncomfortable doing this, so they moved to the back of the line so they could be unattached to everyone else. However, this changed through the duration of the dance activity. Charlie was willing to touch others later on.  *I*: “I’m not super confident in my artwork. It's very vulnerable. And I don't really like being vulnerable, but I tried in this piece.”  *I*: “[I discovered] being able to have privacy but also being able to express myself because I’ve always been scared to do that, but I’m learning more now.” |
| 2. Exploring playfulness and responsibility | *I*: “[During the nature walk session,] I wrote my letter to nature herself. I wrote apologies for all that our species has done. I wrote to them acknowledging that they made us, and we owe them. Writing a letter to something that isn’t a human was interesting. It felt like a plea to a deity. When it went into the river it felt like nature may truly hear me.”  *I*: “[During the nature walk session,] I noticed that there were eagle feathers there by the rocks​. The part when I was skipping rocks felt good, and I just kept on doing and doing and it made me feel relieved. I collected some sea shells.”  *I*: “I had anxiety because I was in a rush. A bit of despair, but [I am] also proud of myself because I have done it.”  *O*: When I checked in on Lake’s painting, it had many colors, and they had created texture with splatters and a sponge. They said they really wanted to draw a noose with frayed edges but didn’t think they could because the guest artist was promoting more abstract art. I [research assistant] said it was up to them how they wanted to represent it. They decided to paint a rope over their background. When we returned from lunch, Lake looked very concerned. They said they were very concerned about being “racist” but were having difficulty matching the paint colors to look like a realistic brown skin color. I wasn’t sure if this meant they were trying to match it to the skin color of one of the participants/volunteers/staff members, but it seemed they just had a certain skin tone in mind and were struggling to create it.  *O*: [During the nature walk session,] Brooklyn challenged me to a rock skipping contest where we each start with 10 life points and lose a life point every time one of us fails to skip a rock. Angel asked to join us. They were singing to Melanie Martinez instrumentals while we were walking outside. Brooklyn asked out loud if they should stop singing and Angel said yes. Brooklyn said nothing can stop them from singing and continued on. Brooklyn made animal-like noises during our time outside; particularly when throwing rocks into the river. |
| 3. Recognizing uniqueness in oneself and others | *I*: “I learned that my art actually can have meaning, you know? Things can actually be represented within what I create. Whenever I’ve made art in the past, people always ask me like ‘Oh, well what does this mean?’ Or ‘what does this represent?’ And I just said ‘I don’t know.’ But it was because I wasn’t thinking about it. [During the camp,] I was more relaxed. I was not being graded on it. I was still sitting next to really talented people, but I didn’t feel stunted. I could still express myself through my art because it was such an individual experience for all of us.”  *I:* “...being around people that were exactly like me but I hadn’t really met before…I felt like I could be weird.”  “Creativity is tied to my internal understanding of myself and also how I try to understand the world.”  *I:* “I am glad I came because people don’t give me any trouble from being the way I am. It felt like I was on eggshells for the first few days purely ‘cause I didn’t know that I could act like myself. Then it felt weird that I wouldn’t get in trouble for taking more food at lunch or needing to be left alone. I was able to be myself ‘cause everyone else was like that too.”  *I:* “It was fun seeing how other people did their art and how other people had their ways of making themselves happy.” |

**Table S15. Participant Six-Month Interviews – Representative Quotations**

| **Core category** | **Representative quotation** |
| --- | --- |
| 1. Flexible approach toward life | “I have noticed that with something that either I have made or that I didn't like, I haven't just thrown it away. I've either tried to fix it or just made something else with it.”  “Last week I was in the gym. I really don't like basketball, but there I was, playing basketball.” |
| 2. Openness to novelty or change | “I've always been in band, always doing music, but I started thinking about it differently and like more creative ways. Instead of just looking at the music and playing what's written, I can kind of interpret it differently.”  “I feel like I have been more active in school and trying new things. I haven't been isolating as much as I used to and doing things that I haven't done before.”  “When I’m around people that I don't know or people older than me, I'm more closed off, but I’ve been around my friends more, and there's one teacher who I know pretty well at my school. So, I've been more authentic towards them because I've kind of opened up to them a little bit.” |
| 3. Expanded view of what it means to be creative | “It definitely made me realize how much everything is a canvas. For example, I myself, my walls, and everything is a canvas.”  “[Creative activities] doesn't have to be about being perfect as long as you're having fun.”  “I realized that creativity is much more fun with limits on it rather than just being like, do whatever you want. I like the structure. If you can bend structure, then that makes it more fun.”  “I had more [creativity] than I originally thought. Other people were more creative than me. They just had more ideas pop up in their head. I didn't think like that, but then, I used their ideas sometimes and came up with my own things. It was collaborative, even if they [peers] didn't know it was.”  “I discovered that I actually am capable of doing things for once. This whole thing [Creativity Camp] was recommended to me by my art teacher in high school because I didn't feel like I could do things correctly. Through that [Creativity Camp], I still have the galaxy painting that we did, and I was like, ‘this actually turned out really well.’ It made me feel better about my abilities. And it made me realize I can do things.”  “Apparently, I have a little bit of a bigger influence on people than I thought I did 'cause like I talked about my vision of snakes, how they're not really what they're represented as. And then the next thing, [camp staff] came up to me, and they were like, I had a dream about you and snakes. I'm like, wow. I have an imprint on people. I guess my brain is way more fascinating to other people than I thought it was.”  “I discovered that it's more fun with other people. It doesn't have to be an individual thing. There are ways to connect your creativity to someone else's. Even doing your own thing side by side, it’s still like having that creative energy together. And some things that you think you're never going to do can still end up being really fun and really useful even if you don't make it a regular thing.” |

**Table S16. Parent/Guardian Interview– Representative Quotations**

| **Core category** | **Representative quotation** |
| --- | --- |
| 1. Expanded personal boundaries | “I got the sense that they felt very safe and comfortable [in the camp] because otherwise they wouldn't have gone back. They mentioned one day that [the Camp staff said,] ‘y'all dancing.’ I was so impressed because I couldn't get them to dance. I thought that was pretty cool that whatever was going on, it made them feel comfortable to try new things.”  “I believe they only ate lunch two other times after that first day. They told me they tried. I praised them for that because they struggled with food and especially eating different things. I'd say, ‘what kind of food was it today?’ [They responded,] ‘Oh, I think it was like curry.’ We talked about how they didn't try that. When I picked them up and if they would say they were hungry, I'd say, ‘What was the lunch today?’ And then they talked about lunch. They were hesitant after that first day. So I would praise them when they told me they tried stuff.”  “They have some significant ones [scars] on their arms. But they weren’t super self conscious to try and hide them in the camp. In the past, if somebody mentioned about their scars, they got withdrawn, and they had a hard time. But they came home and were kind of laughing about a peer in the camp, asking them about their scars. They said ‘[the peer] asked me, ‘what happened, how did you get scars?’ so I looked at them and said I cut myself…duh!’ don’t you know? Why are you asking?’ They weren’t self conscious about it, and they didn't become introverted. It didn't impact their day like it normally would. And then they felt comfortable bringing it up. It wasn't like they felt ashamed. ‘It was just what happened. This is who I am, and you know this, it is what it is like, and you can't change it now.’ They realized that it’s not just them. Others struggle with the same thing.” |
| 2. Enthusiasm through deep engagement | “During the camp, they seemed a lot more motivated and happy and excited. Before those two weeks, they usually took naps during the day and lay around and not have much energy … I noticed a change in their happiness and excitement just about every day. They talked about how they didn’t want it [the camp] to end.”  “They didn’t fight going at all, which tells me they liked it. They would talk to me about what happened [at the camp] which means that they're in a good place and doing fun stuff that they were interested in.”  “I notice more of a sense of willingness and openness. For instance, I said ‘tomorrow, I want to go stand-up paddleboarding’, and they said, ‘oh ok.” Here, I compare it to when they were very depressed. A year ago, it would have been like dragging them to this. So, I feel like there is a little bit more confidence in their ability to withstand the discomfort of a new activity or something different.”  “They really took off on doing origami, like crazy! When they were at residential treatment, I sent them an origami book and a bunch of origami paper and a package that included a bunch of other creative things. And that has sat for a whole year. Nothing touched at all. Now, their desk is entirely covered in origami. So that has been really, a new, kind of thing. And they have been making bracelets again and seem to be finding more satisfaction in their crochet and designing clothing and blankets and a variety of decoration kind of things. Another thing that they have taken up that is new, is their sibling's ukulele and are writing music.  So that is a whole new avenue. There is a combination of a couple things that are new but also a rekindling of some interests.” |

**Table S17. Parent/Guardian Six-Month Interview– Representative Quotations**

| **Core category** | **Representation quotation** |
| --- | --- |
| 1. Sustained enthusiasm | “Creating their own songs, dropping an album are definitely new since the summer [of participating Creativity Camp.] They joined the school [sport] team and went through the season and have been participating in all of them. After school, there's weight training after the season is over. They go to that four days a week. They’ve joined a couple of clubs at school. One of them is like a cultural club surrounding our identification and ethnicity. Another one is an outdoor adventure club, which is way outside of their normal interest or comfort zone. So that was great. And then they’re doing something on the weekends with our [community organization.] That's also a little bit outside of what they would normally have chosen to do. Since the summer, they have been both obviously in action, like all the things I just described, but also rhetorically have been less inclined to say “no” right away. Like almost always like, ‘no, I'm not interested;’ ‘No, I don't want to do that.’ [Instead,] they’re more open to considering and then saying yes and trying new things.” |
| 2. Empowered perspective | “They took a good initiative this past week on confronting someone that's been really bullying them for the last few months. They confronted the problem, went to the dean, spoke to the dean about it, spoke to the counselors about it. And I couldn't see them doing that on their own last year.”  “They did end up going through a partial hospitalization program this year. And I think they were very on board and engaged when they got to that program because by the time they got there, they were like, okay, I'm here for real to learn the skills I need to learn and like they took it pretty seriously. And I think they had some of that experience having done their therapeutic day activities in your group.”  “It was very hard to get them to join in any kind of activity. So the fact that they wanted to and asked to join it [Creativity Camp] was very positive. Since then, they have just been settling into themselves better. They seem to be calmer, having less issues at school and socially. They seem just a little bit lighter… they softened up a little bit. It's not as black and white. It just seems like it's less armor and it's more themselves.” |

1. Fieldnotes are both observational and analytical; and analysis is both interpretative as well as reflective. You will see that your notes are a combination of your observations/descriptions of what is going on in the field, how it is happening, and analytical, how your descriptions link to our inquiry and study questions, how do they differ from or challenge our study questions. Your field notes also show us your own engagement and growth as a researcher. [↑](#footnote-ref-1)
2. Here, and in any other field note, you are telling me the HOW and not the WHY. (The why will come later.) You are showing me and describing to me what "withdrawal" looks like but not necessarily telling me “they are not interested.” Meaning, you are supposed to PAINT A PICTURE IN MIND and DESCRIBE DISINTEREST, etc. [↑](#footnote-ref-2)
3. Fieldnotes are both observational and analytical; and analysis is both interpretative as well as reflective. You will see that your notes are a combination of your observations/descriptions of what is going on in the field, how it is happening, and analytical, how your descriptions link to our inquiry and study questions, how do they differ from or challenge our study questions. Your field notes also show us your own engagement and growth as a researcher. [↑](#footnote-ref-3)
